# Headache among combat-exposed veterans and service members and its relation to mild traumatic brain injury history and other factors: a LIMBIC-CENC study

**DOI:** 10.3389/fneur.2023.1242871

**Published:** 2023-09-20

**Authors:** William C. Walker, Sarah W. Clark, Kaleb Eppich, Elisabeth A. Wilde, Aaron M. Martin, Chelsea M. Allen, Melissa M. Cortez, Mary Jo Pugh, Samuel R. Walton, Kimbra Kenney

**Affiliations:** ^1^Department of Physical Medicine and Rehabilitation (PM&R), School of Medicine, Virginia Commonwealth University, Richmond, VA, United States; ^2^Richmond Veterans Affairs (VA) Medical Center, Central Virginia VA Health Care System, Richmond, VA, United States; ^3^Division of Epidemiology, Department of Internal Medicine, University of Utah, Salt Lake City, UT, United States; ^4^George E. Wahlen VA Salt Lake City Healthcare System, Salt Lake City, UT, United States; ^5^Department of Neurology, Traumatic Brain Injury and Concussion Center, University of Utah, Salt Lake City, UT, United States; ^6^Mental Health and Behavioral Science Service, James A. Haley Veterans’ Hospital, Tampa, FL, United States; ^7^Department of Psychiatry and Behavioral Neurosciences, University of South Florida, Tampa, FL, United States; ^8^Department of Neurology, University of Utah, Salt Lake City, UT, United States; ^9^Informatics, Decision-Enhancement, and Analytic Sciences (IDEAS) Center, Salt Lake City, UT, United States; ^10^Department of Internal Medicine, Division of Epidemiology, Spencer Fox Eccles School of Medicine, University of Utah, Salt Lake City, UT, United States; ^11^Department of Neurology, Uniformed Services University of the Health Sciences, Bethesda, MD, United States

**Keywords:** traumatic brain injury, concussion, headache, postconcussive headache, veterans, blast injuries, military medicine, prediction

## Abstract

**Background:**

Headache (HA) is a common persistent complaint following mild traumatic brain injury (mTBI), but the association with remote mTBI is not well established, and risk factors are understudied.

**Objective:**

Determine the relationship of mTBI history and other factors with HA prevalence and impact among combat-exposed current and former service members (SMs).

**Design:**

Secondary cross-sectional data analysis from the Long-Term Impact of Military-Relevant Brain Injury Consortium—Chronic Effects of Neurotrauma Consortium prospective longitudinal study.

**Methods:**

We examined the association of lifetime mTBI history, demographic, military, medical and psychosocial factors with (1) HA prevalence (“lately, have you experienced headaches?”) using logistic regression and (2) HA burden via the Headache Impact Test-6 (HIT-6) using linear regression. Each lifetime mTBI was categorized by mechanism (blast-related or not) and setting (combat deployed or not). Participants with non-credible symptom reporting were excluded, leaving *N* = 1,674 of whom 81% had positive mTBI histories.

**Results:**

At a median 10 years since last mTBI, mTBI positive participants had higher HA prevalence (69% overall, 78% if 3 or more mTBIs) and greater HA burden (67% substantial/severe impact) than non-TBI controls (46% prevalence, 54% substantial/severe impact). In covariate-adjusted analysis, HA prevalence was higher with greater number of blast-related mTBIs (OR 1.81; 95% CI 1.48, 2.23) non-blast mTBIs while deployed (OR 1.42; 95% CI 1.14, 1.79), or non-blast mTBIs when not deployed (OR 1.23; 95% CI 1.02, 1.49). HA impact was only higher with blast-related mTBIs. Female identity, younger age, PTSD symptoms, and subjective sleep quality showed effects in both prevalence and impact models, with the largest mean HIT-6 elevation for PTSD symptoms. Additionally, combat deployment duration and depression symptoms were factors for HA prevalence, and Black race and Hispanic/Latino ethnicity were factors for HA impact. In sensitivity analyses, time since last mTBI and early HA onset were both non-significant.

**Conclusion:**

The prevalence of HA symptoms among formerly combat-deployed veterans and SMs is higher with more lifetime mTBIs regardless of how remote. Blast-related mTBI raises the risk the most and is uniquely associated with elevated HA burden. Other demographic and potentially modifiable risk factors were identified that may inform clinical care.

## Introduction

Headache (HA) is an important worldwide health problem, with HA disorders (>5 stereotypical HA episodes per year) ranked as the second leading cause of years lived with disability (1). HA is also a common sequela of traumatic brain injury (TBI) in both civilian (2) and military (3) populations. Although HA can occur after any severity of TBI, the focus of this study is HA among persons with mild TBI (mTBI), which accounts for well over 80% of TBI events.

Prior longitudinal studies show HA is a common persistent complaint following mTBI in both military (4, 5) and civilian (6) populations. HA disorders in patients with mTBI may or may not meet the criteria to be termed “posttraumatic” HA. Specifically, the International Classification of Headache Disorders-3 (ICHD-3) classifies HA after TBI as a secondary HA disorder when the initial HA onset or exacerbation of pre-existing headache begins within 7 days following trauma or injury, or within 7 days after recovering consciousness and/or within 7 days after recovering the ability to sense and report pain (7). Posttraumatic HA is considered to be persistent if it lasts beyond 3 months after injury, which is the commonly accepted timeframe for transition from acute to persistent or chronic HA from any condition, including as a primary disorder. The classification of posttraumatic HA is progressively more challenging to determine as the TBI event becomes more remote (e.g., years later). Acute or persisting posttraumatic HA may resolve over time, but another HA may later emerge for which the TBI may not be a contributing factor. Accordingly, almost all existing research on HA in the very chronic phase of TBI does not attempt to make the distinction between trauma-related HA and HA from other viable sources (8).

Risk factors for having HA in either the acute or chronic phase after TBI are not well understood (8, 9). Intuitively, greater severity of TBI would presume greater risk for HA; however, research has not clearly demonstrated this relation. Some studies paradoxically show greater prevalence of HA after mTBI compared to moderate or severe (mod-sev) TBI (9); however, these studies may be biased with over-representation of patients with mTBI seeking medical care for high symptom levels, including HA, versus the majority who rapidly recover and are not included in these studies. Large studies that have examined the prevalence of HA remotely after mod-sev TBI have not demonstrated an association with TBI severity indices such as posttraumatic amnesia (PTA) duration and HA (2, 3). Given this, there remains significant debate about the extent of any late effect, including HA, that is potentially attributable to a remote TBI alone versus other factors and comorbidities, especially those related to mental health.

Beyond TBI severity, risk factors for HA after TBI have been primarily examined with respect to acute predictors (8). For the chronic phase of TBI, the most frequently cited risk factors include female sex and history of HA disorder prior to TBI, especially migraine type (10), in both patients with mTBI (11) and mod-sev TBI (2, 12). Findings on differences related to age are mixed, with some studies showing younger age as a risk factor (13, 14). Other factors associated with poorer HA outcomes include lower education, learning disabilities, sleep difficulties, lifestyle factors (e.g., alcohol use), self-efficacy, and resilience. In the military population, combat deployment itself was identified as a risk factor for HA disorders, although TBI history was not assessed (15). Another aspect is the relationship between HA symptoms and other active health conditions, particularly mental health. Posttraumatic stress disorder (PTSD) and depression symptoms among military service members (SMs) have been associated with post-deployment HA (15, 16). Studies in the civilian population have also demonstrated an association between PTH and PTSD, anxiety, and depression (9, 10). Despite these and other investigations, a recent systematic review concluded that there are no identified evidence-based risk factors for HA (8) in the chronic phase after TBI and that further studies are warranted.

Thus, there is an evidence gap related to the scope of the HA problem among persons with previous mTBI (s) in both military and civilian populations. Better information is needed regarding HA prevalence and risk factors to inform clinical care, including targeted screening and monitoring strategies and the identification and treatment of modifiable co-morbidities that are associated with increased HA prevalence. The objectives of this study were to (1) describe the prevalence and impact of HA among combat-exposed current and former SMs with varied mTBI histories (2), assess the unique contribution of mTBI history on their remote (mean >10 years after mTBI) HA prevalence and impact of current headache on daily life activities, and (3) examine the effects of other factors on remote HA prevalence and impact.

## Methods

### Design

Using a cross-sectional design, this study analyzed LIMBIC-CENC Prospective Longitudinal Study (PLS) enrollment data. Detailed information on methods, including aims, recruitment procedures, eligibility of the LIMBIC-CENC PLS project is available elsewhere (17, 18). In brief, the LIMBIC-CENC PLS is a large multi-center longitudinal, observational study of current and former United States SMs with combat exposure, with both an established cohort (baseline) and ongoing enrollment (prospective, longitudinal) at 11 sites located across the country. The primary objective is to better understand the long-term neurologic effects of combat exposures in general and mTBI in particular, and their interrelationships with other aspects of health. During the baseline evaluation, all participants completed a comprehensive assessment including face-to-face structured interviews, self-reported questionnaires, extensive neuropsychological testing, biometrics, and many other tests not used for the current analyses. For the self-reported questionnaires, most participants completed them in-person in a quiet office space on either paper copies or a web-based application. Research staff only intervened if paper form completion was incomplete, to request the mark any missing response (s). If the participant questioned research staff on interpretation of any item then they were instructed to use their best judgement based on the scripted instructions from the validated instrument. To shorten the time of their in-person visit, some participants chose to complete their self-reported questionnaires at home on the internet or by mailing or bring in paper versions. The LIMBIC-CENC PLS, including creation of a database registry and all secondary analyses, was approved by the local Institutional Review Boards at each enrollment site.

### Participants

LIMBIC-CENC PLS participants have variable lifetime mTBI histories, ranging from entirely negative to over 10 prior mTBIs. To be eligible, individuals were required to be 18 years of age or older and have a history of combat exposure. The only exclusions were a history of mod-sev TBI or a major neurological or psychiatric disorder that significantly impaired functioning. Individuals with other common mental health conditions, such as depression or PTSD, were included. All participants provided written consent prior to any study procedures.

**Figure 1:**
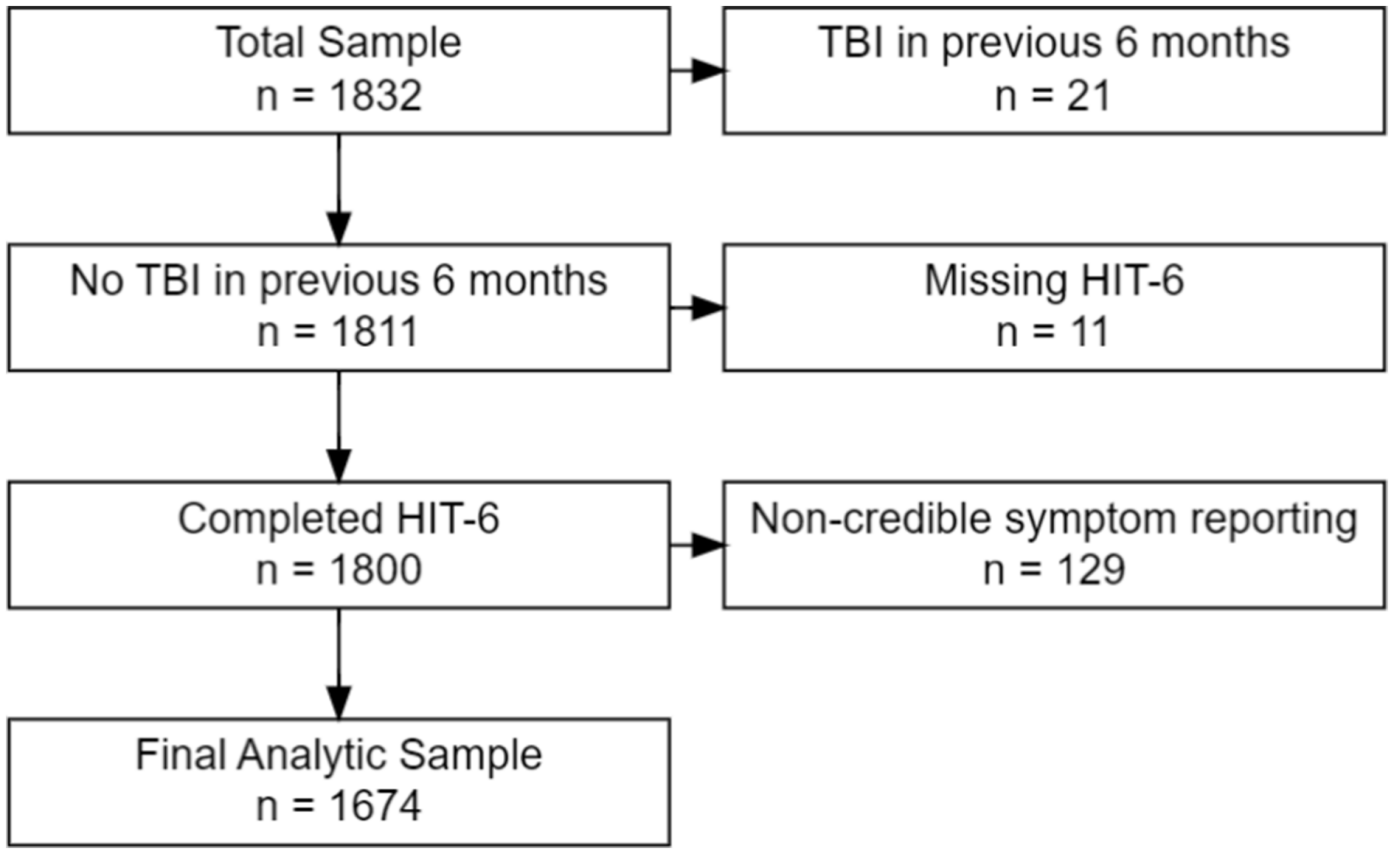
Study sample inclusion flow diagram.

For this secondary analysis, all LIMBIC-CENC PLS participants whose enrollment (baseline) assessment data were available at time of dataset extraction were included (*n* = 1,832). Because the current study focus was HA in the chronic phase of mTBI, we excluded individuals who had sustained an mTBI within 6 months prior to enrollment (*n* = 21) or missing data (*n* = 11) on the HA outcome measure. We also excluded participants with evidence of noncredible symptom reporting based on failing (126) the Mild Brain Injury Atypical Symptom (mBIAS) scale, a validated self-reported measure of symptom reporting credibility in the mTBI population using the developer’s recommended cut-point of 8 or higher (19). This left a final analytic sample of 1,674 participants (see Figure 1).

### Measures

#### Lifetime mild TBI history

Clinical diagnosis of mTBI was assessed via a multi-step process centered on a structured face-to-face interview. The first step was to identify and catalog every potential concussive events (PCEs) across each participant’s lifetime using a modified version of a validated TBI screening interview, the Ohio State University TBI Identification (OSU TBI-ID) (20) to. PCEs were then assessed more thoroughly with a validated structured interview tool, the Virginia Commonwealth University retrospective Concussion Diagnostic Interview (VCU rCDI) (21) which has an algorithm that generates a preliminary TBI diagnosis. The algorithm-generated diagnosis was then reviewed by the site principal investigator, checked against available medical records, and further reviewed by a centralized quality assurance process that included an expert committee to determine a final clinical diagnosis according to VA/DoD definition of mTBI (22). This diagnosis also adheres to the American Congress of Rehabilitation Medicine criteria for definition of mTBI (23). Based on the VCU rCDI interview information, each positive mTBI was categorized by environmental context: sustained during a combat deployment (combat mTBI) versus other time of life (non-combat mTBI), mechanism (blast-related versus blunt-only), and presence of early onset HA after TBI (within 2 weeks). We also examined time since last mTBI for the current analyses.

#### Headache point prevalence and impact (primary outcomes)

The Headache Impact Test (HIT-6) is a validated 6-item self-reported questionnaire of HA burden on daily functioning during the past 4 weeks (24). Total scores for impact range from 36 to 72, and levels can be interpreted as little or no impact (49 or less), some impact (50–55), substantial impact (56–59), and severe impact (60–78). The HIT-6 does include one item (item #1) that directly queries pain intensity (“when you have headache, how often is your pain severe?”) We also included a stem question “lately, have you experienced headaches?” that was used as a point prevalence estimate. If participants asked how “lately” was defined, they were instructed to use their own judgement.

#### Basic demographics

Age and self-identity of gender, race, and ethnicity were collected at baseline by Behavioral Risk Factor Surveillance System (BRFSS) a self-reported questionnaire developed the Centers for Disease Control and Prevention (25).

#### Military exposures

Total months combat-deployed was calculated by summing the duration of every military combat deployment ascertained by military records. Combat intensity was measured by Section D of the Deployment Risk and Resiliency Inventory, Version 2 (DRRI-2), a self-reported questionnaire of military combat exposures (26). During the LIMBIC-CENC PCE/TBI structured interview, participants were also queried on the number of controlled blast exposures they were exposed to during lifetime.

#### Psychosocial factors

The Posttraumatic Stress Disorder Checklist for DSM-5 (PCL-5) is a 20-item self-report questionnaire measure of post-traumatic stress disorder (PTSD) symptoms including re-experiencing, avoidance and numbing, hyperarousal, and negative cognitions and mood (27) over the past month. Higher scores reflect greater symptom severity. The Patient Health Questionnaire (PHQ-9) assessed self-reported depression symptoms over the past 2 weeks (28). Scores range from 0 (none) to 27 (severe). Alcohol use (no alcohol use, non-hazardous use, versus hazardous use) was determined from the Alcohol Use Disorder Identification Test (AUDIT-C), a self-reported questionnaire (29). Post-deployment social support was assessed with the Deployment Risk and Resiliency Inventory, Version 2 (DRRI-2) Section O (DRRI-2-O), a 10-item self-reported questionnaire on the extent to which family, friends, coworkers, employers, and community provide emotional sustenance and instrumental assistance (26). Finally, self-efficacy was ascertained by the general self-efficacy (GSE) scale, a self-reported questionnaire with a higher total score indicating greater self-efficacy (30).

#### Medical comorbidities

Using the forementioned BRFSS self-reported questionnaire, we collected self-reported hypertension, hyperlipidemia, diabetes, stroke, and other neurological disorders. Subjective sleep quality over the past month was evaluated with the Pittsburgh Sleep Quality Index (PSQI), (31), another self-reported questionnaire. Sleep apnea symptoms were assessed via a modified version of the STOP-BANG self-reported questionnaire, with high risk classified by scores greater than or equal to three (32). Obesity categories were created by directly measuring height and weight to then calculate body mass index (BMI).

### Statistical methods

The relationship between the HA prevalence outcome (lately, have you experienced headache?) stratified by groups based on number of historical lifetime mTBIs (none, 1–2 mTBI, and 3+ mTBIs) was first assessed using Pearson’s chi-squared test. For those subjects who disclosed experiencing HA lately, HIT-6 total score, HIT-6 impact categories, and HIT-6 item #1 were summarized using mean (standard deviation, SD) or counts and percentages. These variables were compared across mTBI history groups using the Kruskal–Wallis rank sum test for HIT-6 total score because of the ordered nature of the history groups and Pearson’s chi-squared test for HIT-6 total score impact categories and HIT-6 item #1 responses.

Pairwise chi-squared tests were used to examine all possible paired comparisons between mTBI groups and HIT-6 stem question as well as HIT-6 impact categories. Dunn’s test was used for pairwise comparisons between mTBI groups and HIT-6 total score. All *post hoc* analyses used Bonferroni’s correction to account for inflated type I error from multiple testing. Clinical and demographic characteristics were then reported using mean (SD) and median (interquartile range, IQR) for continuous variables and counts and percentages for categorical variables. These variables were stratified by whether they endorsed experiencing HA lately and compared across groups using the Wilcoxon rank sum test for continuous variables and Pearson’s chi-squared test or Fisher’s exact test for categorical variables.

We analyzed the prevalence outcome (experiencing HA lately) using univariable and multivariable logistic regression because it is a binary (yes/no) variable. Among the subjects who endorsed experiencing HA lately, linear regression was used with for the HA impact outcome (HIT-6 total score) because it is a continuous variable. For all models, assumptions such as linearity, normality, etc. were assessed to verify that the model was appropriate to use. Variables that were selected to be included in the models were either primary or secondary variables of interest—blast and mTBI related variables—or covariates selected using a combination of clinical judgment and those that were significantly different between headache groups in bivariate analysis at the 0.05 level. For consistency, we used the same variables for the separate logistic and linear regressions. For cumulative number of lifetime mTBIs, we used three variables: number of blast-related mTBIs, number of blunt combat mTBIs, and number of blunt non-combat mTBIs. This was done to include mTBI context and mechanism together without overlap and because only 1% of blast-related mTBIs occurred outside of deployment during a military training accident. Continuous variables included were scaled in order to compare the 75th percentile to the 25th percentile in both models. Beta coefficients and odds ratios (OR) were reported with 95% confidence intervals (CI) and corresponding *p*-values.

Additionally, variables relevant only to TBI positive participants (early HA after TBI; time since last TBI) were examined in separate sensitivity analysis excluding the TBI negative group.

All analyses were performed using R version 4.2.1. Statistical significance was assessed at the 0.05 level and all tests were two-tailed.

## Results

In our final sample of 1,674 combat-exposed current and former SMs, 19% had an entirely negative lifetime mTBI history, 47% had sustained 1-2 mTBIs, and 34% had 3 or more. Rates of positive history across themTBI mechanism/setting categories were 63% for Combat mTBI(s), 67% for Non-combat mTBI(s), and 37% for Blast-related mTBI(s).

### HA prevalence and impact across mTBI history groups (0, 1–2, 3+)

Within our entire sample, the point prevalence of endorsing yes to “lately, have you experienced headache?” was 65% at time of enrollment. In general HA was significantly more prevalent and more impactful with higher number of lifetime mTBIs. Specifically, HA prevalence was 46% for those with no prior TBI, 63% for 1–2 lifetime mTBIs, and 78% for 3+ lifetime mTBIs (see Table 1).

**Table 1:** Headache (HA) prevalence (experienced HA lately) stratified by # lifetime mTBIs.

	No TBI	1-2 mTBIs	3+ mTBIs	Total	p-value^a^
HA Lately					**<0.001**
No	173 (54%)	292 (37%)	125 (22%)	590 (35%)	
Yes	148 (46%)	504 (63%)	432 (78%)	1,084 (65%)	
Total	321 (100%)	796 (100%)	557 (100%)	1,674 (100%)	

a Pearson’s chi-squared test; *p*-values bolded if <0.05.

Among the participants endorsing HA lately, the HIT-6 total scores, the impact level categories, and HIT-6 item #1 responses also all differed by the number of lifetime mTBI groups (see Table 2). For all three measures, the 3-group difference was driven by lower symptoms for the negative TBI group compared to the two positive mTBI groups, with no difference between the 1–2 mTBI and 3+ mTBIs groups in post-hoc pairwise comparisons.For example, the rate of severe HA pain sometimes, often or always was 70% for the no TBI group compared to 78% for those with 1-2 or 3+ lifetime mTBIs. (see Table 3 for HIT-6 item #1 post-hoc testing; the other post-hoc testing data are available in Supplementary Tables S1, S2).

**Table 2:** Headache (HA) impact stratified by # lifetime mTBIs.

Characteristic	**All**, *N* = 1,084	**No TBI**, *N* = 148	**1-2 mTBIs**, *N* = 504	**3+ mTBIs**, *N* = 432	***p*-value** ^1^
HIT-6 Total Score, Mean (SD)	58.6 (8.8)	56.3 (9.4)	59.1 (8.9)	58.8 (8.4)	**0.005**
HIT-6 Impact Categories, n (%)					**0.012**
Little/None	167 (15%)	37 (25%)	73 (14%)	57 (13%)	
Some	212 (20%)	30 (20%)	100 (20%)	82 (19%)	
Substantial	162 (15%)	19 (13%)	68 (13%)	75 (17%)	
Severe	542 (50%)	61 (41%)	263 (52%)	218 (50%)	
Headache Severe Pain, n (%)					**0.007**
Never	22 (2.0%)	9 (6.1%)	7 (1.4%)	6 (1.4%)	
Rarely	226 (21%)	35 (24%)	103 (20%)	88 (20%)	
Sometimes	448 (41%)	55 (37%)	198 (39%)	195 (45%)	
Very Often	314 (29%)	37 (25%)	163 (32%)	114 (26%)	
Always	73 (6.7%)	11 (7.5%)	33 (6.5%)	29 (6.7%)	

^1^Kruskal-Wallis rank sum test for HIT-6 total score; Pearson's Chi-squared test for HIT-6 and Severe Pain frequency categories; p-value bolded if < 0.05

**Table 3:** Post-hoc comparisons of HIT-6 headache severity categories by # lifetime mTBIs.

Dimension	Value	Never	Rarely	Sometimes	Very Often	Always
No TBI	Residuals	3.782071	0.9440547	−1.046433	−1.098988	0.38620065
No TBI	*P* values	**0.002333**	1.00	1.00	1.00	1.00
1-2 mTBIs	Residuals	−1.398384	−0.3259913	−1.297286	2.265382	−0.23624680
1-2 mTBIs	*P* values	1.00	1.00	1.00	0.352338	1.00
3+ mTBIs	Residuals	−1.220961	−0.3282617	2.053412	−1.538916	−0.02948149
3+mTBIs	*P* values	1.00	1.00	0.600490	1.00	1.00

*p-value bolded if <0.05.*

### Sample characteristics and bivariate relationships to prevalence of HA lately

Characteristics of the cohort to be examined as covariates are displayed in Table 4 for categorical variables and Table 5 for continuous variables, with the overall sample in the left-hand column and stratified by HA “lately” negative and positive groups in the right-hand columns. Unadjusted bivariate relationships with HA lately prevalence were assessed between each characteristic with differences at *p* < 0.05 bolded. The type and number of every type of lifetime mTBI was different between groups, as was time since last TBI, early HA after TBI, gender, ethnicity, age, number of controlled blast exposures, combat intensity, combat deployment time, alcohol use, obstructive sleep apnea risk level, symptomatology of depression, PTSD, and sleep quality, as well as social support and self-efficacy.

**Table 4:** Categorical covariates stratified by absence/presence of headache (HA).

**Characteristic**	Overall		Experienced HA Lately		***p*-value** ^2^
	***N* = 1,674** ^1^	***N* Missing**	**No**, *N* = 590^1^	**Yes**, *N* = 1,084^1^	
Gender		1			**<0.001**
Male	1,458 (87%)		547 (93%)	911 (84%)	
Female	215 (13%)		43 (7.3%)	172 (16%)	
Race		11			0.6
White	1,224 (74%)		441 (75%)	783 (73%)	
Black or African American	307 (18%)		99 (17%)	208 (19%)	
American Indian or Alaska Native	15 (0.9%)		4 (0.7%)	11 (1.0%)	
Asian	26 (1.6%)		7 (1.2%)	19 (1.8%)	
Other	91 (5.5%)		34 (5.8%)	57 (5.3%)	
Ethnicity		20			**0.005**
Not Hispanic or Latino	1,372 (83%)		506 (86%)	866 (81%)	
Hispanic or Latino	282 (17%)		79 (14%)	203 (19%)	
Blast TBI	606 (36%)	0	117 (20%)	489 (45%)	**<0.001**
Non-blast TBI	1,192 (71%)	0	385 (65%)	807 (74%)	**<0.001**
Deploy TBI	889 (53%)	0	201 (34%)	688 (63%)	**<0.001**
Non-deploy TBI	1,087 (65%)	0	357 (61%)	730 (67%)	**0.005**
Early HA after TBI*	379 (28%)	0	88 (21%)	291 (31%)	**<0.001**
Controlled blast exposures		0			**0.017**
None	464 (28%)		179 (30%)	285 (26%)	
Minimal (1-9)	422 (25%)		159 (27%)	263 (24%)	
Light (10-29)	272 (16%)		100 (17%)	172 (16%)	
Moderate (30-98)	228 (14%)		72 (12%)	156 (14%)	
Heavy (99+)	288 (17%)		80 (14%)	208 (19%)	
Alcohol Use (AUDIT-C)		6			**0.019**
None	300 (18%)		91 (16%)	209 (19%)	
Moderate	788 (47%)		268 (46%)	520 (48%)	
Risky	580 (35%)		228 (39%)	352 (33%)	
PCL-5/PTSD		8			**<0.001**
No PTSD (≤35)	1,171 (70%)		499 (85%)	672 (62%)	
Possible PTSD (36-49)	289 (17%)		60 (10%)	229 (21%)	
Highly probable PTSD (≥50)	206 (12%)		28 (4.8%)	178 (16%)	
PHQ-9/Depression		18			**<0.001**
No depression (0-4)	598 (36%)		334 (57%)	264 (25%)	
Mild depression (5-9)	485 (29%)		146 (25%)	339 (32%)	
Moderate depression (10-15)	384 (23%)		85 (15%)	299 (28%)	
Moderate/severe depression (≥16)	189 (11%)		21 (3.6%)	168 (16%)	
BMI category		11			0.093
<20	18 (1.1%)		5 (0.9%)	13 (1.2%)	
>29	875 (53%)		289 (49%)	586 (54%)	
20-29	770 (46%)		292 (50%)	478 (44%)	
HTN	581 (35%)	0	187 (32%)	394 (36%)	0.076
Stroke	8 (0.5%)	0	2 (0.3%)	6 (0.6%)	0.8
Neuro Disorder	71 (4.2%)	0	24 (4.1%)	47 (4.3%)	>0.9
Diabetes	91 (5.4%)	0	32 (5.4%)	59 (5.4%)	>0.9
OSA high risk (STOP-BANG)	313 (19%)	23	82 (14%)	231 (22%)	**<0.001**

^1^n (%)

^2Pearson's Chi-squared test; Fisher's exact test; p-value bolded if < 0.05^

*N=1,353 with positive mTBI histories

**Table 5:** Continuous covariates stratified by absence/presence of Headache (HA).

**Characteristic**	Overall		Experienced HA Lately		***p*-value** ^1^
	***N* = 1,674**	***N* Missing**	**No**, *N* = 590	**Yes**, *N* = 1,084	
Age (years)		0			**0.039**
Mean (SD)	41 (10)		42 (11)	40 (9)	
Median (IQR)	39 (33, 48)	0	40 (32, 51)	39 (33, 47)	
Num of lifetime mTBIs					**<0.001**
Mean (SD)	2.15 (1.97)		1.58 (1.69)	2.45 (2.04)	
Median (IQR)	2.00 (1.00, 3.00)		1.00 (0.00, 2.00)	2.00 (1.00, 3.00)	
Time since last TBI (years)*		0			**<0.001**
Mean (SD)	12 (9)		14 (11)	11 (8)	
Median (IQR)	10 (6, 14)		11 (7, 18)	9 (5, 13)	
Num of non-blast TBIs overall		0			**<0.001**
Mean (SD)	1.61 (1.66)		1.33 (1.48)	1.76 (1.73)	
Median (IQR)	1.00 (0.00, 2.00)		1.00 (0.00, 2.00)	1.00 (0.00, 3.00)	
Num non-blast TBIs when deployed		0			**<0.001**
Mean (SD)	0.35 (0.63)		0.23 (0.50)	0.42 (0.69)	
Median (IQR)	0.00 (0.00, 1.00)		0.00 (0.00, 0.00)	0.00 (0.00, 1.00)	
Num non-blast TBIs not deployed		0			**<0.001**
Mean (SD)	1.26 (1.42)		1.11 (1.31)	1.35 (1.46)	
Median (IQR)	1.00 (0.00, 2.00)		1.00 (0.00, 2.00)	1.00 (0.00, 2.00)	
Num of months combat deployed		34			**<0.001**
Mean (SD)	20 (13)		18 (12)	21 (13)	
Median (IQR)	15 (11, 26)		14 (10, 24)	17 (12, 28)	
Combat Intensity (DRRI-2)		3			**<0.001**
Mean (SD)	37 (15)		33 (13)	39 (15)	
Median (IQR)	34 (24, 48)		30 (22, 40)	37 (26, 50)	
Num of controlled blasts		0			**0.001**
Mean (SD)	28 (37)		23 (34)	30 (38)	
Median (IQR)	7 (0, 45)		5 (0, 30)	9 (0, 50)	
Depression (PHQ9)		18			**<0.001**
Mean (SD)	7.7 (5.9)		5.0 (4.9)	9.2 (5.8)	
Median (IQR)	7.0 (3.0, 11.0)		4.0 (1.0, 8.0)	8.0 (5.0, 13.0)	
PTSD (PCL5)		8			**<0.001**
Mean (SD)	25 (19)		17 (16)	30 (18)	
Median (IQR)	23 (9, 39)		12 (3, 25)	28 (15, 43)	
Sleep Quality (PSQI)		28			**<0.001**
Mean (SD)	10.2 (4.8)		7.9 (4.5)	11.4 (4.4)	
Median (IQR)	10.0 (6.0, 14.0)		8.0 (4.0, 11.0)	12.0 (8.0, 15.0)	
Social Support (DRRI-2)		2			**<0.001**
Mean (SD)	39 (8)		40 (8)	38 (8)	
Median (IQR)	40 (34, 45)		42 (36, 47)	39 (33, 44)	
Self-Efficacy (GSE)		3			**<0.001**
Mean (SD)	32.1 (4.8)		33.3 (4.5)	31.4 (4.8)	
Median (IQR)	32.0 (29.0, 36.0)		34.0 (30.0, 37.0)	31.0 (28.0, 35.0)	

^1^Wilcoxon rank sum test; p-value bolded if < 0.05

*Time since last mTBI only applies to participants with positive mTBI histories (N=1,353)

### Main multivariable regression analyses

Results of the main logistic regression for Experiencing HA Lately (at the time of enrollment) are displayed in Table 6 showing odds ratios (OR), confidence interval (CI) and *p*-values. For TBI history, the number of lifetime mTBIs of every type was significant, including blast-related (OR = 1.80), Blunt during combat-deployment (OR = 1.41), and Blunt outside of deployment (OR = 1.23). Other significant factors included identifying as female (OR = 3.57), age (0.76), total months combat-deployed (OR = 1.23), and symptoms of depression on PHQ-9 (OR = 1.56), PTSD on PCL-5 (OR = 1.54), and disturbed sleep quality on PSQI (OR = 1.78).

**Table 6:** Multivariable logistic regression—experience headaches lately yes/no.

**Characteristic**	Multivariable		
	**OR** ^1^	**95% CI** ^2^	***p*-value** ^3^
Gender			
Male	—	—	
Female	3.57	2.37, 5.48	**<0.001**
Num of blast TBIs (combat and noncombat)	1.80	1.47, 2.22	**<0.001**
Num of combat/nonblast TBIs	1.41	1.13, 1.78	**0.003**
Num of noncombat/nonblast TBIs	1.23	1.02, 1.49	**0.035**
Num of months combat deployed	1.23	1.04, 1.45	**0.014**
Controlled blast exposures			
None	—	—	
Minimal (1-9)	1.06	0.76, 1.48	0.7
Light (10-29)	0.98	0.67, 1.44	>0.9
Moderate (30-98)	1.23	0.81, 1.88	0.3
Heavy (99+)	1.10	0.72, 1.66	0.7
OSA high risk (STOP-BANG)	1.13	0.80, 1.62	0.5
Race			
White	—	—	
Black or African American	0.93	0.67, 1.29	0.7
American Indian or Alaska Native	2.35	0.57, 16.1	0.3
Asian	2.63	1.06, 7.22	**0.046**
Other	0.53	0.30, 0.95	**0.030**
Ethnicity			
Not Hispanic or Latino	—	—	
Hispanic or Latino	1.33	0.93, 1.92	0.12
Alcohol Use (AUDIT-C)			
None	—	—	
Moderate	1.27	0.89, 1.81	0.2
Risky	0.87	0.60, 1.26	0.5
HTN			
No	—	—	
Yes	1.20	0.92, 1.57	0.2
Age	0.76	0.62, 0.93	**0.009**
BMI categories			
20-29	—	—	
<20	1.00	0.31, 3.62	>0.9
>29	1.03	0.79, 1.33	0.8
Depression (PHQ-9 total score)	1.56	1.13, 2.15	**0.007**
PTSD (PCL-5 total score)	1.54	1.07, 2.23	**0.021**
Sleep Quality Disturbance (PSQI total score)	1.78	1.40, 2.28	**<0.001**
Social Support (DRRI-2 social total)	1.15	0.95, 1.40	0.2
Combat Intensity (DRRI-2 combat total)	1.09	0.83, 1.42	0.5
Self-Efficacy (GSE total)	1.08	0.87, 1.35	0.5

^1^OR = Odds Ratio (expressed as 75^th^ versus 25^th^ percentile for continuous variables)

^2^CI = Confidence Interval

^3^p-value bolded if < 0.05

Results of the linear regression for HA impact measured by HIT-6 total score among participants experiencing HA lately are displayed in Table 7. For TBI history, only blast-related mTBIs were significant (Beta 0.57). Blunt-only mTBIs did not reach significance, regardless of contextual type (combat or non-combat). Other factors found significant in the HIT-6 linear regression that were also significant in the HA prevalence logistic regression were female identity (Beta 3.4), younger age (Beta −0.98), PTSD symptoms (Beta 4.9), and reduced sleep quality (Beta 1.4). Demographic characteristics that were significant in the HIT-6 score linear regression model but not the preceding HA prevalence model were Black racial identity (Beta 2.3) and Hispanic/Latino ethnic identity (Beta 2.0) as compared with White/non-Hispanic racial/ethnic identity. Additionally, risky alcohol use was associated with lower HIT-6 total scores (Beta −2.3) compared to non-drinkers. Overall, Multiple *R*^2^ for the model was 0.350, indicating the model accounted for 35% the variance in HIT-6 total score.

**Table 7:** Multivariable linear regression for HIT-6 total score (multiple *R*^2^ = 0.350).

**Characteristic**	Multivariable		
	**Beta** ^1^	**95% CI** ^2^	***p*-value** ^3^
Gender			
Male	—	—	
Female	3.4	2.1, 4.8	**<0.001**
Num of blast TBIs (combat and noncombat)	0.57	0.02, 1.1	**0.043**
Num of combat/nonblast TBIs	0.38	−0.32, 1.1	0.3
Num of noncombat/nonblast TBIs	−0.09	−0.75, 0.58	0.8
Num of months combat deployed	−0.01	-0.59, 0.57	>0.9
Controlled blast exposures			
None	—	—	
Minimal (1-9)	−0.98	−2.3, 0.33	0.14
Light (10-29)	−0.32	−1.8, 1.2	0.7
Moderate (30-98)	−0.71	−2.3, 0.86	0.4
Heavy (99+)	−0.71	−2.2, 0.81	0.4
OSA high risk (STOP-BANG)	0.69	−0.54, 1.9	0.3
Race			
White	—	—	
Black or African American	2.3	1.1, 3.6	**<0.001**
American Indian or Alaska Native	2.9	−1.5, 7.2	0.2
Asian	−1.0	−4.4, 2.4	0.6
Other	2.4	0.28, 4.5	**0.027**
Ethnicity			
Not Hispanic or Latino	—	—	
Hispanic or Latino	2.0	0.79, 3.3	**0.001**
Alcohol Use (AUDIT-C)			
None	—	—	
Moderate	-0.52	−1.8, 0.75	0.4
Risky	−2.3	−3.6, -0.91	**0.001**
HTN			
No	—	—	
Yes	0.53	−0.47, 1.5	0.3
Age	−0.98	−1.8, -0.12	**0.026**
BMI categories			
20-29	—	—	
<20	0.56	−3.6, 4.8	0.8
>29	−0.33	−1.3, 0.67	0.5
Depression (PHQ-9 total score)	0.67	−0.39, 1.7	0.2
PTSD (PCL-5 total)	4.9	3.6, 6.2	**<0.001**
Sleep Quality Disturbance (PSQI total score)	1.4	0.48, 2.3	**0.003**
Social Support (DRRI-2 social total)	0.24	−0.47, 0.94	0.5
Combat Intensity (DRRI-2 combat total)	0.34	−0.61, 1.3	0.5
Self-Efficacy (GSE total score)	−0.70	−1.5, 0.09	0.084

^1^Beta expressed as 75th versus 25th percentile for continuous variables.

^2^CI = Confidence Interval.

^3^p-value bolded if < 0.05.

### Multivariable regression sensitivity analyses for mTBI-positive participants only

When excluding the TBI negative participants, time since last mTBI was not significant (*p* > 0.05) in either the logistic regression prevalence or linear regression HA impact models, nor was HA onset within 2 weeks of mTBI (full sensitivity analysis results are available in Supplementary Tables S3, S4).

## Discussion

This study provides valuable empirical data on the prevalence, risk factors and impact of HA among previously combat-deployed SMs and veterans. The overall sample (*n* = 1,674), which included 19% with negative TBI histories, had a HA point prevalence (i.e., HA lately) of 65%. Even though mTBI (s) were mostly very remote, with a median 10 years since last mTBI, a greater number of mTBIs was associated with higher HA prevalence, reaching 78% for participants with 3 or more mTBIs (see Table 1). Additionally, a greater number of lifetime mTBIs was associated with more impactful HA when HA was endorsed (see Table 2). For example, 67% showed substantial or severe impact on HIT-6 when mTBI history was positive in contrast to 54% when negative.

A unique aspect of our study was to examine current HA burden in relation to military-relevant subtypes of mTBI history including mechanism (blast-related or not) and setting (combat-deployed or not). This was done while also adjusting for and examining many other potential HA contributors including demographic, military, medical, and psychological factors. Thus, our findings provide additional insights into how HA impact may vary by mechanism and setting that are unique to SMs and veterans and that are independent of PTSD, depression, sleep quality and self-efficacy. The covariate-adjusted logistic regression model for HA prevalence (see Table 6) showed higher prevalence with a greater number of any subtype of mTBI (see Table 6), with the nominally highest OR for blast-related mechanism (OR 1.80; 95% CI 1.47, 2.22). Broadly, these findings provide strong empirical evidence that lifetime mTBI history is an independent risk factor for chronic HA symptoms. They are also consistent with Hoge et al. (33) who showed that HA was the main symptom linked to combat mTBI history at an earlier timepoint after adjusting for similar factors including PTSD. Prior work examining less rigorous ICD coding among combat veterans has also demonstrated an increased risk of HA diagnosis codes with respect to mTBI history codes when adjusting for psychiatric condition codes.

In our covariate adjusted models for HA impact (see Table 7), the most striking finding was greater impact for blast-related mTBI but not for blunt-only mTBI. On average, the HIT-6 total score was 0.6 points higher for each additional blast mTBI. In contrast, blunt-only mTBIs were not associated with higher HIT-6 scores in our adjusted analyses, in either deployed or non-deployed setting. This finding, together with the nominally higher odds for HA prevalence after blast mTBI (see Table 6), suggests that veterans and SMs with blast-related mTBI may have unique susceptibility to chronic HA problems. Animal model research has identified vascular pathology and inflammatory changes unique to blast-TBI, and if translatable to humans may contribute to the poorer HA outcomes after blast-related mTBI. Our finding of greater HA impact for blast-related mTBI has some parallel with a prior study in the warrior strong cohort study (*n* = 1,074) showing that soldiers with posttraumatic HA (*n* = 198) had greater headache complexity (*p* < 0.001) compared to non-concussed soldiers (*n* = 647) (34), but they did not specifically examine blast mechanism. It is also worth noting again that we observed elevated risk at very remote time points and even after adjusting for concurrent symptom measures including PTSD, depression, sleep quality and self-efficacy.

Surprisingly, our sensitivity analysis (see Supplementary Tables S3, S4) restricted to mTBI positive participants showed that neither the self-report of early onset HA after mTBI (within 2 weeks) nor time since last mTBI was associated with either outcome, HA prevalence or HA impact, when adjusting for other covariates. The non-association with early onset HA suggests that the HA in our sample on average does not meet the definition of PTHA *per se*, however our study design which lacked acute data could not directly examine this question. The lack of change over time must be interpreted with the caveat that we only included participants who were greater than 6 months since their last mTBI, so all were already in the “chronic” stage. This non-association does suggest that post-acute HA in this population does not fade over time, and that there is a potential unmet care need.

Regarding other covariates, our study indicates that combat deployment duration itself is a unique risk factor for later HA prevalence (see Table 6), suggesting a contribution to a general physiologic and/or psychologic stress exposure that chronically increases HA risk. This finding has also been demonstrated in prior research (15), and may help explain why the OR for HA prevalence in our study was higher if the blunt-only mTBI was sustained during deployment compared to some other time of life. A potential explanation for these findings is that the additional stressors of combat deployment interfere with early recovery from mTBI, increasing the odds of late effects such as HA disorders.

Our large sample, which included 215 females (13%), enabled us to examine their relative risk for HA, a previously understudied research question in the military population due to insufficient numbers of females in most prior HA studies. Our results show that female sex had the nominally highest OR (3.57; 2.37, 5.48) for experiencing HA lately (see Table 6), and had a strong association with higher HA impact (Beta 3.4; 2.1, 4.8; see Table 7). These findings are consistent with the literature from the general population indicates that females are not only at higher risk for experiencing general pain (35), but are also at higher risk for HA, especially migraine-type (36). Given the high rate of migraine type HA that has been demonstrated after TBI (3, 6, 9, 37), a potential mechanistic explanation for the risk elevation for female individuals in our study is related to unmasking a genetic predisposition (38) and/or hormonally-mediated increases in calcitonin gene-related peptide levels (39). Younger age was found to be associated with both HA prevalence and HA impact in our study (see Tables 6, 7), and this is also similar to studies of HA in the general population. In our study, Black racial identity and Hispanic/Latino ethnicity were uniquely associated with higher HA impact compared to White/non-Hispanic participants (see Table 7). These demographic findings suggest that individuals representing marginalized or historically excluded groups (individuals identifying as female, Black, or Hispanic/Latino) may benefit from treatment programs that use targeted outreach strategies, address access barriers, and include appropriate patient education materials.

Symptom measures examined as covariates including depression, PTSD, and sleep quality, were significantly associated with prevalence of experiencing HA lately (see Table 6). PTSD and sleep quality were also associated with HA impact (see Table 7), with higher PTSD symptom endorsement (75th versus 25th percentile on PCL-5) showing the nominally strongest relationship to HIT-6 of any covariate (Beta 4.9; 3.6, 6.2). Thus, combat-exposed military personnel with PTSD are at greatest risk for having their HA contribute to severe negative life impact. Given the concurrent data collection for all of these symptom measures, the pathway of these relationship cannot be determined. However, a bidirectional relationship seems most plausible, as has been demonstrated between migraine and depression in the general population (40). Additionally, sleep quality is likely to both impact and be impacted by HA presence and severity of HA impact, as individuals may engage in compensatory behaviors (e.g., napping) in response to HA-related sleep disturbances, which are detrimental to nighttime sleep quality, and ultimately lower headache threshold (41).

These symptom measure covariate findings demonstrate how HA could complicate and interact with other aspects of outcome in this population. These also represent modifiable targets for treatment. Treatment implications include the potential benefit of cognitive behavioral therapy for HA in this population. The findings also support a holistic approach to caring for persons with chronic HA after TBI that addresses all potential modifiable factors, including medical-based management of headache and mood disorders along with psycho-behavioral management. Future longitudinal analysis could provide further insights into treatment by elucidating the directional pathways of these associations.

A seemingly paradoxical finding in our study was a reduced HA impact for alcohol use at a risky level verses abstinence (see Table 7). This is consistent with a systematic review showing that people with migraine HA consume less alcohol than peers (42). While it is possible that alcohol use is a protective factor, it may be that alcohol use patterns are measuring one or more latent trait variables that may better explain this association. Support for this comes from prior research on alcohol often showing an asymmetrical U-shaped relationship with a variety of health outcomes in the general population. For HA in particular, data identifying alcohol as a trigger for migraine is limited (42), with a recent large study showing no association (43).

For other exposures or medical factors, covariates with no significance in either model included hypertension, hyperlipidemia, obstructive sleep apnea risk level, body mass index, combat intensity, and number of controlled blast exposures. Psychosocial variables with negative findings in both models included social support and general self-efficacy.

### Study strengths

Study strengths included our large sample (*n* = 1,674) of individuals with military combat exposure drawn from the LIMBIC-CENC multicenter cohort with rigorously determined lifetime mTBI histories and a large breadth of data available from their comprehensive assessments. The sample was also diverse racially (19% Black identity) and ethnically (17% Hispanic or Latino identify). The inclusion of non-TBI comparators and incorporation of sociodemographic and symptom measures allowed us to better determine the unique contribution of their prior mild TBIs on current HA burden. The inclusion of military-relevant mTBI classification also allowed us to parse out the effects of blast-related mechanism and combat-deployed setting. By demonstrating TBI and other risk factors for HA prevalence and HA impact in this population and showing the association with psychological functioning, our study also highlights an opportunity to advance clinical care and patient outcomes.

#### Study limitations

A limitation of this study was the use of self-report questionnaires for most of the measures including mental health comorbidities and the HA outcome. Other study limitations with respect to the HA outcome included a lack of information on pre-morbid HA, a previously shown predictor of HA after TBI (2), and on premorbid migraine and psychological health with both previously shown predictors of persistent PTHA (10). We also lacked that data differentiating continued HA persistence versus *de novo* onset long after mTBI. We did collect and include HA symptoms within 2 weeks of mTBI as a covariate, which had no significant effect t in either the HA prevalence or the HA impact regression model. Thus, the HA prevalence associations with remote combat and non-combat mTBIs and HA impact association with combat mTBIs we found suggest a risk elevation that does not fit criteria for the current diagnostic term of posttraumatic HA as per The International Classification of Headache Disorders (ICHD) (7): HA onset (or worsening of premorbid HA) “within 7 days following trauma or injury, or within 7 days after recovering consciousness and/or within 7 days after recovering the ability to sense and report pain” (8). Regardless, we cannot determine if the HA reported may have persisted since injury or developed at some period of time after injury in relation to other factors or co-occurring health conditions. Our assessment of headache did not include data on specific quality, location, duration, and additional features (e.g., photophobia, nausea) of headaches that would allow further investigation between differences in headache type (e.g., tension-type, migraine-type, cervicogenic, mixed). Further, prior research has shown medication overuse is associated with persistence of PTHA (10) and some participants may had HA resolution with proper treatment, but we did not examine the potential mediating effect of medications and other treatments received. Lastly, although we used a validated structured interview method with layers of quality assurance, the retrospective identification of historical mTBI is prone to recall bias. More recent mild TBI diagnosis criteria guidelines include the use of blood biomarkers (44), an emerging area of research which so far is only validated for acute head CT decision making with respect to complicated mild TBI with associated intracerebral hemorrhage (45).

#### Implications for future research

Future research is needed to better understand the relationships we found between HA and these other symptom measures and to study effective treatments and behavioral interventions for HA after mTBI. Future research is also recommended to examine in greater depth factors that may be contributing to the increased HA susceptibility among female SM and veterans. Longitudinal research could offer more insights on causal or directional pathways, especially for the psychologic factors. Our finding of worse HA burden after blast-related mTBI suggests that individuals with blast-related mTBI may need a different type of clinical care pathway and/or treatments compared to blunt-only mTBI, with further research needed including studies examining the mechanism underlying this association. Potential mechanisms may include damage to head and neck tissues resulting in neurogenic inflammation, hyperexcitability of peripheral nociceptors, chronic allodynia/hyperalgesia, damage to spinothalamic/thalamocortical pathways, damage to or dysfunction of pain-inhibition pathways, and/or vascular contributors, including dysregulation of pericranial, intracranial and dural arteries (46).

## Conclusion

Experiencing recent HA is extremely common among formerly combat-exposed military personnel and is associated with substantial to severe negative impact on life quality. We demonstrated that remote mTBI history is associated with elevated odds of HA and a higher degree of HA impact, especially for mTBIs that were blast-related. This was shown in both bivariate analyses and multivariable regression adjusting for numerous sociodemographic, health, and symptom measures – including PTSD. These findings highlight the ramifications of mTBI in the military population, and will inform clinical screening, education, and monitoring strategies. Clinical strategies to provide early or targeted intervention should also incorporate the other risk factors we identified among our covariates including female sex, Black racial identity, Hispanic/Latino ethnicity, and younger age. Modifiable treatment targets we identified include PTSD, depression, and sleep quality.

## Data availability statement

The datasets presented in this study can be found in online repositories. The names of the repository/repositories and accession number(s) can be found at: Federal Interagency Traumatic Brain Injury Research (FITBIR) Informatics System. https://fitbir.nih.gov/.

## Ethics statement

The studies involving humans were approved by Local IRB at every participating site as well as US Department of Defense HRPO. The studies were conducted in accordance with the local legislation and institutional requirements. The participants provided their written informed consent to participate in this study. Written informed consent was obtained from the individual(s) for the publication of any potentially identifiable images or data included in this article.

## Author contributions

WW: conceptualization, methodology, investigation, data interpretation, and writing, reviewing and editing. SC: conceptualization, data interpretation, and writing, reviewing and editing. KE: statistical methods, data curation, data analysis, data interpretation, and writing, reviewing and editing. EW, AM, MC, and MP: methodology and writing, reviewing and editing. CA: data analysis, data interpretation, and reviewing and editing. SW: reviewing and editing. KK: conceptualization, methodology, data interpretation, and writing, reviewing and editing. All authors contributed to the article and approved the submitted version.

## Funding

This work was supported by the Assistant Secretary of Defense for Health Affairs endorsed by the Department of Defense, through the Psychological Health/Traumatic Brain Injury Research Program Long-Term Impact of Military-Relevant Brain Injury Consortium (LIMBIC) Award/W81XWH-18-PH/TBIRP-LIMBIC under Awards No. W81XWH1920067 and W81XWH-13-2-0095, and by the U.S. Department of Veterans Affairs Awards No. I01 CX002097, I01 CX002096, I01 HX003155, I01 RX003444, I01 RX003443, I01 RX003442, I01 CX001135, I01 CX001246, I01 RX001774, I01 RX 001135, I01 RX 002076, I01 RX 001880, I01 RX 002172, I01 RX 002173, I01 RX 002171, I01 RX 002174 I01 RX 002170, and I01 CX001820. The U.S. Army Medical Research Acquisition Activity, 839 Chandler Street, Fort Detrick MD 21702-5014 is the awarding and administering acquisition office. Additional funding was provided by VA Health Services Research and Development (IK6HX002608). This investigation was also supported by the University of Utah Study Design and Biostatistics Center, with funding in part from the National Center for Research Resources and the National Center for Advancing Translational Sciences, National Institutes of Health, through Grant UL1TR002538 (formerly 5UL1TR001067-05, 8UL1TR000105, and UL1RR025764).

## Conflict of interest

The authors declare that the research was conducted in the absence of any commercial or financial relationships that could be construed as a potential conflict of interest.

## Publisher’s note

All claims expressed in this article are solely those of the authors and do not necessarily represent those of their affiliated organizations, or those of the publisher, the editors and the reviewers. Any product that may be evaluated in this article, or claim that may be made by its manufacturer, is not guaranteed or endorsed by the publisher.

## Author disclaimer

The views, opinions, interpretations, conclusions and recommendations expressed in this manuscript are those of the authors and do not reflect the official policy of the Department of the Navy, Department of the Army, Department of Defense, Department of Veterans Affairs or the U.S. Government.

## References

[1] Saylor D Steiner TJ. The global burden of headache. Semin Neurol. (2018) 38:182–90. doi: 10.1055/s-0038-1646946 29791944

[2] Hoffman JM Lucas S Dikmen S Braden CA Brown AW Brunner R. Natural history of headache after traumatic brain injury. J Neurotrauma. (2011) 28:1719–25. doi: 10.1089/neu.2011.1914, PMID: 21732765 PMC3172878

[3] Walker WC Seel RT Curtiss G Warden DL. Headache after moderate and severe traumatic brain injury: a longitudinal analysis. Arch Phys Med Rehabil. (2005) 86:1793–800. doi: 10.1016/j.apmr.2004.12.042, PMID: 16181945

[4] Theeler BJ Erickson JC. Posttraumatic headache in military personnel and veterans of the Iraq and Afghanistan conflicts. Curr Treat Options Neurol. (2012) 14:36–49. doi: 10.1007/s11940-011-0157-2, PMID: 22116663

[5] Theeler BJ Flynn FG Erickson JC. Headaches after concussion in US soldiers returning from Iraq or Afghanistan. Headache. (2010) 50:1262–72. doi: 10.1111/j.1526-4610.2010.01700.x 20553333

[6] Lucas S Hoffman JM Bell KR Dikmen S. A prospective study of prevalence and characterization of headache following mild traumatic brain injury. Cephalalgia. (2014) 34:93–102. doi: 10.1177/0333102413499645 23921798

[7] International Headache Society (IHS). Headache Classification Committee of the International Headache Society (IHS) The International Classification of Headache Disorders 3rd edition. Cephalalgia. (2018) 38:1–211. doi: 10.1177/0333102417738202, PMID: 29368949

[8] Andersen AM Ashina H Iljazi A Al-Khazali HM Chaudhry B Ashina M. Risk factors for the development of post-traumatic headache attributed to traumatic brain injury: a systematic review. Headache. (2020) 60:1066–75. doi: 10.1111/head.13812, PMID: 32320055

[9] Ashina H Porreca F Anderson T Amin FM Ashina M Schytz HW. Post-traumatic headache: epidemiology and pathophysiological insights. Nat Rev Neurol. (2019) 15:607–17. doi: 10.1038/s41582-019-0243-8, PMID: 31527806

[10] Chan TLH Woldeamanuel YW. Exploring naturally occurring clinical subgroups of post-traumatic headache. J Headache Pain. (2020) 21:12. doi: 10.1186/s10194-020-1080-2, PMID: 32033526 PMC7006085

[11] Terry DP Reddi PJ Cook NE Seifert T Maxwell BA Zafonte R. Acute effects of concussion in youth with pre-existing migraines. Clin J Sport Med. (2021) 31:430–7. doi: 10.1097/JSM.0000000000000791, PMID: 31842054

[12] Walker WC Marwitz JH Wilk AR Ketchum JM Hoffman JM Brown AW. Prediction of headache severity (density and functional impact) after traumatic brain injury: a longitudinal multicenter study. Cephalalgia. (2013) 33:998–1008. doi: 10.1177/0333102413482197, PMID: 23575819

[13] Cancelliere C Boyle E Côté P Holm LW Salmi L-R Cassidy JD. Development and validation of a model predicting post-traumatic headache six months after a motor vehicle collision in adults. Accid Anal Prev. (2020) 142:105580. doi: 10.1016/j.aap.2020.105580, PMID: 32445970

[14] Stacey A Lucas S Dikmen S Temkin N Bell KR Brown A. Natural history of headache five years after traumatic brain injury. J Neurotrauma. (2017) 34:1558–64. doi: 10.1089/neu.2016.4721, PMID: 27927072

[15] Jankosky CJ Hooper TI Granado NS Scher A Gackstetter GD Boyko EJ. Headache disorders in the millennium cohort: epidemiology and relations with combat deployment. Headache. (2011) 51:1098–111. doi: 10.1111/j.1526-4610.2011.01914.x, PMID: 21675968

[16] Afari N Harder LH Madra NJ Heppner PS Moeller-Bertram T King C. PTSD, combat injury, and headache in veterans returning from Iraq/Afghanistan. Headache. (2009) 49:1267–76. doi: 10.1111/j.1526-4610.2009.01517.x 19788469

[17] Walker WC Carne W Franke LM Nolen T Dikmen SD Cifu DX. The Chronic Effects of Neurotrauma Consortium (CENC) multi-centre observational study: description of study and characteristics of early participants. Brain Inj. (2016) 30:1469–80. doi: 10.1080/02699052.2016.1219061, PMID: 27834538

[18] Walker WC Hirsch S Carne W Nolen T Cifu DX Wilde EA. Chronic Effects of Neurotrauma Consortium (CENC) multicentre study interim analysis: differences between participants with positive versus negative mild TBI histories. Brain Inj. (2018) 32:1079–89. doi: 10.1080/02699052.2018.1479041, PMID: 29851515

[19] Cooper DB Nelson L Armistead-Jehle P Bowles AO. Utility of the mild brain injury atypical symptoms scale as a screening measure for symptom over-reporting in operation enduring freedom/operation iraqi freedom service members with post-concussive complaints. Arch Clin Neuropsychol. (2011) 26:718–27. doi: 10.1093/arclin/acr070, PMID: 21873326

[20] Corrigan JD Bogner J. Initial reliability and validity of the Ohio State University TBI Identification Method. The J Head Trauma Rehabil (2007) 22:318–29. doi: 10.1097/01.HTR.0000300227.67748.77 18025964

[21] Walker WC Cifu DX Hudak AM Goldberg G Kunz RD Sima AP. Structured interview for mild traumatic brain injury after military blast: inter-rater agreement and development of diagnostic algorithm. J Neurotrauma. (2015) 32:464–73. doi: 10.1089/neu.2014.3433, PMID: 25264909

[22] Management of Concussion/mTBI Working Group. VA/DoD Clinical Practice Guideline for Management of Concussion/Mild Traumatic Brain Injury. J Rehabil Res Develop. (2009) 46:CP1-68. 20108447

[23] American Congress of Rehabilitation Medicine, M. T. B. I. C. Definition of mild traumatic brain injury. J Head Trauma Rehabil. (1993) 8:86–87.

[24] Kosinski M Bayliss MS Bjorner JB Ware JE Garber WH Batenhorst A. A six-item short-form survey for measuring headache impact: The HIT-6. Qual Life Res An Int J Qual Life Aspects Treat Care Rehabil. (2003) 12:963–74. doi: 10.1023/a:1026119331193 14651415

[25] Rolle-Lake L Robbins E. Behavioral Risk Factor Surveillance System. In: StatPearls. (StatPearls Publishing). (2021). 31971707

[26] Vogt D Smith BN King LA King DW Knight J Vasterling JJ. Deployment risk and resilience inventory-2 (DRRI-2): an updated tool for assessing psychosocial risk and resilience factors among service members and veterans. J Trauma Stress. (2013) 26:710–7. doi: 10.1002/jts.21868, PMID: 24490250

[27] Blevins CA Weathers FW Davis MT Witte TK Domino JL. The Posttraumatic Stress Disorder Checklist for DSM-5 (PCL-5): Development and Initial Psychometric Evaluation. J Trauma Stress (2015) 28:489–98. doi: 10.1002/jts.22059 26606250

[28] Kroenke K Spitzer RL Williams JB. The PHQ-9: Validity of a brief depression severity measure. J Gen Int Med. (2001) 16:606–13. doi: 10.1046/j.1525-1497.2001.016009606.x PMC1495268 11556941

[29] Kuitunen-Paul S Roerecke M. Alcohol use disorders identification test (AUDIT) and mortality risk: a systematic review and meta-analysis. J Epidemiol Community Health. (2018) 72:856–63. doi: 10.1136/jech-2017-210078, PMID: 29921648

[30] Schwarzer R Renner B. Social-cognitive predictors of health behavior: action self-efficacy and coping self-efficacy. Health Psychol. (2000) 19:487–95. doi: 10.1037/0278-6133.19.5.487, PMID: 11007157

[31] Buysse DJ Reynolds CF Monk TH Berman SR Kupfer DJ. The Pittsburgh Sleep Quality Index: A new instrument for psychiatric practice and research. Psychiatry Res. (1989) 28:193–213. doi: 10.1016/0165-1781(89)90047-4 2748771

[32] Chung F Abdullah HR Liao P. STOP-Bang Questionnaire: A Practical Approach to Screen for Obstructive Sleep Apnea. Chest (2016) 149:631–38. doi: 10.1378/chest.15-0903 26378880

[33] Hoge CW McGurk D Thomas JL Cox AL Engel CC Castro CA. Mild Traumatic Brain Injury in U.S. Soldiers Returning from Iraq. New Eng J Med. (2008) 358:453–63. doi: 10.1056/NEJMoa072972 18234750

[34] Scher AI McGinley JS Wirth RJ Lipton RB Terrio H Brenner LA. Headache complexity (number of symptom features) differentiates post-traumatic from non-traumatic headaches. Cephalalgia. (2021) 41:582–92. doi: 10.1177/0333102420974352, PMID: 33242991

[35] Beaudoin FL Kessler RC Hwang I Lee S Sampson NA AURORA Study Group. Pain after a motor vehicle crash: The role of socio-demographics, crash characteristics and peri-traumatic stress symptoms. Euro J Pain. (2021) 25:1119–136. doi: 10.1002/ejp.1733 PMC10913946 33458880

[36] Tsai C-K Tsai C-L Lin G-Y Yang F-C Wang S-J. Sex differences in chronic migraine: focusing on clinical features, pathophysiology, and treatments. Curr Pain Headache Rep. (2022) 26:347–55. doi: 10.1007/s11916-022-01034-w, PMID: 35218478

[37] Hoffman JM Lucas S Dikmen S Temkin N. Clinical perspectives on headache after traumatic brain injury. PM R. (2020) 12:967–74. doi: 10.1002/pmrj.12338, PMID: 32003524

[38] Dias A Mariz T Sousa A Lemos C Alves-Ferreira M. A review of migraine genetics: gathering genomic and transcriptomic factors. Hum Genet. (2022) 141:1–14. doi: 10.1007/s00439-021-02389-7, PMID: 34686893

[39] Raffaelli B Storch E Overeem LH Terhart M Fitzek MP Lange KS. Sex hormones and calcitonin gene-related peptide in women with migraine: a cross-sectional, matched cohort study. Neurology. (2023) 100:e1825–35. doi: 10.1212/WNL.0000000000207114, PMID: 36813730 PMC10136010

[40] Jahangir S Adjepong D Al-Shami HA Malik BH. Is there an association between migraine and major depressive disorder? A narrative review. Cureus. (2020) 12:e8551. doi: 10.7759/cureus.8551, PMID: 32670688 PMC7357317

[41] Lovati C D’Amico D Raimondi E Mariani C Bertora P. Sleep and headache: a bidirectional relationship. Expert Rev Neurother. (2010) 10:105–17. doi: 10.1586/ern.09.135 20021325

[42] Panconesi A. Alcohol and migraine: trigger factor, consumption, mechanisms. A review. J Headache Pain. (2008) 9:19–27. doi: 10.1007/s10194-008-0006-1, PMID: 18231712 PMC3476173

[43] Vives-Mestres M Casanova A Puig X Ginebra J Rosen N. Alcohol as a trigger of migraine attacks in people with migraine. Results from a large prospective cohort study in English-speaking countries. Headache. (2022) 62:1329–38. doi: 10.1111/head.14428, PMID: 36437596 PMC10099573

[44] Silverberg ND Iverson GL Cogan A Dams-O’Connor K Delmonico R Graf MJP. The American Congress of Rehabilitation Medicine diagnostic criteria for mild traumatic brain injury. Arch Phys Med Rehabil. (2023) 104:1343–55. doi: 10.1016/j.apmr.2023.03.036, PMID: 37211140

[45] Bazarian JJ Biberthaler P Welch RD Lewis LM Barzo P Bogner-Flatz V. Serum GFAP and UCH-L1 for prediction of absence of intracranial injuries on head CT (ALERT-TBI): a multicentre observational study. Lancet Neurol. (2018) 17:782–9. doi: 10.1016/S1474-4422(18)30231-X, PMID: 30054151

[46] Defrin R. Chronic post-traumatic headache: clinical findings and possible mechanisms. J Man Manip Ther. (2014) 22:36–43. doi: 10.1179/2042618613Y.0000000053, PMID: 24976746 PMC4062350

