# Corrigendum: Headache among combat-exposed veterans and service members and its relation to mild TBI history and other factors: a LIMBIC-CENC study

**DOI:** 10.3389/fneur.2025.1577480

**Published:** 2025-04-16

**Authors:** William C. Walker, Sarah W. Clark, Kaleb Eppich, Elisabeth A. Wilde, Aaron M. Martin, Chelsea M. Allen, Melissa M. Cortez, Mary Jo Pugh, Samuel R. Walton, Kimbra Kenney

**Affiliations:** ^1^Department of Physical Medicine and Rehabilitation (PM&R), School of Medicine, Virginia Commonwealth University, Richmond, VA, United States; ^2^Richmond Veterans Affairs (VA) Medical Center, Central Virginia VA Health Care System, Richmond, VA, United States; ^3^Division of Epidemiology, Department of Internal Medicine, University of Utah, Salt Lake City, UT, United States; ^4^George E. Wahlen VA Salt Lake City Healthcare System, Salt Lake City, UT, United States; ^5^Department of Neurology, Traumatic Brain Injury and Concussion Center, University of Utah, Salt Lake City, UT, United States; ^6^Mental Health and Behavioral Science Service, James A. Haley Veterans' Hospital, Tampa, FL, United States; ^7^Department of Psychiatry and Behavioral Neurosciences, University of South Florida, Tampa, FL, United States; ^8^Department of Neurology, University of Utah, Salt Lake City, UT, United States; ^9^Informatics, Decision-Enhancement, and Analytic Sciences (IDEAS) Center, Salt Lake City, UT, United States; ^10^Department of Internal Medicine, Division of Epidemiology, Spencer Fox Eccles School of Medicine, University of Utah, Salt Lake City, UT, United States; ^11^Department of Neurology, Uniformed Services University of the Health Sciences, Bethesda, MD, United States

**Keywords:** traumatic brain injury, concussion, headache, postconcussive headache, veterans, blast injuries, military medicine, prediction

In the published article, there was an error in “Figure 1. Study Sample Inclusion Flow Diagram”. After publication, the authors were informed that one site's IRB retroactively deemed their data to be invalid, and therefore cannot be published (Site #10; Eisenhower Army Medical Center; located near Augusta, GA). The updated figure reflects changes excluding the removed data and updated the results data in all main Tables and supplementary Tables; Table 1. Headache (HA) prevalence (experienced HA lately) stratified by # lifetime mTBIs; Table 2. Headache (HA) impact stratified by # lifetime mTBIs; Table 3. *Post-hoc* comparisons of HIT-6 headache severity categories by # lifetime mTBIs; Table 4. Categorical covariates stratified by absence/presence of Headache (HA); Table 5. Continuous covariates stratified by absence/presence of Headache (HA); Table 6. Multivariable logistic regression—experience headaches lately yes/no; Table 7. Multivariable linear regression for HIT-6 total score (multiple *R*^2^ = 0.350).

**Table 1:** Headache (HA) prevalence (experienced HA lately) stratified by # lifetime mTBIs.

	**No TBI**	**1-2 mTBIs**	**3+ mTBIs**	**Total**	***p*-value^a^**
HA lately					**< 0.001**
No	173 (54%)	292 (37%)	125 (22%)	590 (35%)	
Yes	148 (46%)	504 (63%)	432 (78%)	1,084 (65%)	
Total	321 (100%)	796 (100%)	557 (100%)	1,674 (100%)	

^a^Pearson's Chi-squared test; *p*-values bolded if < 0.05.

**Table 2:** Headache (HA) impact stratified by # lifetime mTBIs.

**Characteristic**	**All, *N* = 1,084**	**No TBI, *N* = 148**	**1-2 mTBIs, *N* = 504**	**3+ mTBIs, *N* = 432**	***p*-value^a^**
HIT-6 total score, mean (SD)	58.6 (8.8)	56.3 (9.4)	59.1 (8.9)	58.8 (8.4)	**0.005**
HIT-6 impact categories, *n* (%)					**0.012**
Little/none	167 (15%)	37 (25%)	73 (14%)	57 (13%)	
Some	212 (20%)	30 (20%)	100 (20%)	82 (19%)	
Substantial	162 (15%)	19 (13%)	68 (13%)	75 (17%)	
Severe	542 (50%)	61 (41%)	263 (52%)	218 (50%)	
Headache severe pain, *n* (%)					**0.007**
Never	22 (2.0%)	9 (6.1%)	7 (1.4%)	6 (1.4%)	
Rarely	226 (21%)	35 (24%)	103 (20%)	88 (20%)	
Sometimes	448 (41%)	55 (37%)	198 (39%)	195 (45%)	
Very often	314 (29%)	37 (25%)	163 (32%)	114 (26%)	
Always	73 (6.7%)	11 (7.5%)	33 (6.5%)	29 (6.7%)	

^a^Kruskal-Wallis rank sum test for HIT-6 total score; Pearson's Chi-squared test for HIT-6 and severe pain frequency categories; *p*-value bolded if < 0.05.

**Table 3:** *Post-hoc* comparisons of HIT-6 headache severity categories by # lifetime mTBIs.

**Dimension**	**Value**	**Never**	**Rarely**	**Sometimes**	**Very often**	**Always**
No TBI	Residuals	3.782071	0.9440547	−1.046433	−1.098988	0.38620065
No TBI	*P* values	**0.002333**	1.00	1.00	1.00	1.00
1–2 mTBIs	Residuals	−1.398384	−0.3259913	−1.297286	2.265382	−0.23624680
1–2 mTBIs	*P* values	1.00	1.00	1.00	0.352338	1.00
3+ mTBIs	Residuals	−1.220961	−0.3282617	2.053412	−1.538916	−0.02948149
3+mTBIs	*P* values	1.00	1.00	0.600490	1.00	1.00

*p-*value bolded if < 0.05.

**Table 4:** Categorical covariates stratified by absence/presence of Headache (HA).

	**Overall**		**Experienced HA lately**		
**Characteristic**	***N*** = **1,674**^a^	***N*** **Missing**	**No, N** = **590**^a^	**Yes**, ***N*** = **1,084**^a^	***p*** **-value** ^b^
Gender		1			**< 0.001**
Male	1,458 (87%)		547 (93%)	911 (84%)	
Female	215 (13%)		43 (7.3%)	172 (16%)	
Race		11			0.6
White	1,224 (74%)		441 (75%)	783 (73%)	
Black or African American	307 (18%)		99 (17%)	208 (19%)	
American Indian or Alaska Native	15 (0.9%)		4 (0.7%)	11 (1.0%)	
Asian	26 (1.6%)		7 (1.2%)	19 (1.8%)	
Other	91 (5.5%)		34 (5.8%)	57 (5.3%)	
Ethnicity		20			**0.005**
Not Hispanic or Latino	1,372 (83%)		506 (86%)	866 (81%)	
Hispanic or Latino	282 (17%)		79 (14%)	203 (19%)	
Blast TBI	606 (36%)	0	117 (20%)	489 (45%)	**< 0.001**
Non-blast TBI	1,192 (71%)	0	385 (65%)	807 (74%)	**< 0.001**
Deploy TBI	889 (53%)	0	201 (34%)	688 (63%)	**< 0.001**
Non-deploy TBI	1,087 (65%)	0	357 (61%)	730 (67%)	**0.005**
Early HA after TBI^*^	379 (28%)	0	88 (21%)	291 (31%)	**< 0.001**
Controlled blast exposures		0			**0.017**
None	464 (28%)		179 (30%)	285 (26%)	
Minimal (1–9)	422 (25%)		159 (27%)	263 (24%)	
Light (10–29)	272 (16%)		100 (17%)	172 (16%)	
Moderate (30–98)	228 (14%)		72 (12%)	156 (14%)	
Heavy (99+)	288 (17%)		80 (14%)	208 (19%)	
Alcohol use (AUDIT-C)		6			**0.019**
None	300 (18%)		91 (16%)	209 (19%)	
Moderate	788 (47%)		268 (46%)	520 (48%)	
Risky	580 (35%)		228 (39%)	352 (33%)	
PCL-5/PTSD		8			**< 0.001**
No PTSD (≤ 35)	1,171 (70%)		499 (85%)	672 (62%)	
Possible PTSD (36-49)	289 (17%)		60 (10%)	229 (21%)	
Highly probable PTSD (≥50)	206 (12%)		28 (4.8%)	178 (16%)	
PHQ-9/depression		18			**< 0.001**
No depression (0–4)	598 (36%)		334 (57%)	264 (25%)	
Mild depression (5–9)	485 (29%)		146 (25%)	339 (32%)	
Moderate depression (10–15)	384 (23%)		85 (15%)	299 (28%)	
Moderate/severe depression (≥16)	189 (11%)		21 (3.6%)	168 (16%)	
BMI category		11			0.093
< 20	18 (1.1%)		5 (0.9%)	13 (1.2%)	
>29	875 (53%)		289 (49%)	586 (54%)	
20-29	770 (46%)		292 (50%)	478 (44%)	
HTN	581 (35%)	0	187 (32%)	394 (36%)	0.076
Stroke	8 (0.5%)	0	2 (0.3%)	6 (0.6%)	0.8
Neuro disorder	71 (4.2%)	0	24 (4.1%)	47 (4.3%)	>0.9
Diabetes	91 (5.4%)	0	32 (5.4%)	59 (5.4%)	>0.9
OSA high risk (STOP-BANG)	313 (19%)	23	82 (14%)	231 (22%)	**< 0.001**

^a^n (%).

^s^Pearson's Chi-squared test; Fisher's exact test;

*p*-value bolded if < 0.05.

^*^N = 1,353 with positive mTBI histories.

**Table 5:** Continuous covariates stratified by absence/presence of Headache (HA).

	**Overall**		**Experienced HA lately**		
**Characteristic**	***N*** = **1,674**	***N*** **Missing**	**No**, ***N*** = **590**	**Yes**, ***N*** = **1,084**	***p*** **-value** ^a^
Age (years)		0			**0.039**
Mean (SD)	41 (10)		42 (11)	40 (9)	
Median (IQR)	39 (33, 48)	0	40 (32, 51)	39 (33, 47)	
Num of lifetime mTBIs					**< 0.001**
Mean (SD)	2.15 (1.97)		1.58 (1.69)	2.45 (2.04)	
Median (IQR)	2.00 (1.00, 3.00)		1.00 (0.00, 2.00)	2.00 (1.00, 3.00)	
Time since last TBI (years)^*^		0			**< 0.001**
Mean (SD)	12 (9)		14 (11)	11 (8)	
Median (IQR)	10 (6, 14)		11 (7, 18)	9 (5, 13)	
Num of non-blast TBIs overall		0			**< 0.001**
Mean (SD)	1.61 (1.66)		1.33 (1.48)	1.76 (1.73)	
Median (IQR)	1.00 (0.00, 2.00)		1.00 (0.00, 2.00)	1.00 (0.00, 3.00)	
Num non-blast TBIs when deployed		0			**< 0.001**
Mean (SD)	0.35 (0.63)		0.23 (0.50)	0.42 (0.69)	
Median (IQR)	0.00 (0.00, 1.00)		0.00 (0.00, 0.00)	0.00 (0.00, 1.00)	
Num non-blast TBIs not deployed		0			**< 0.001**
Mean (SD)	1.26 (1.42)		1.11 (1.31)	1.35 (1.46)	
Median (IQR)	1.00 (0.00, 2.00)		1.00 (0.00, 2.00)	1.00 (0.00, 2.00)	
Num of months combat deployed		34			**< 0.001**
Mean (SD)	20 (13)		18 (12)	21 (13)	
Median (IQR)	15 (11, 26)		14 (10, 24)	17 (12, 28)	
Combat intensity (DRRI-2)		3			**< 0.001**
Mean (SD)	37 (15)		33 (13)	39 (15)	
Median (IQR)	34 (24, 48)		30 (22, 40)	37 (26, 50)	
Num of controlled blasts		0			**0.001**
Mean (SD)	28 (37)		23 (34)	30 (38)	
Median (IQR)	7 (0, 45)		5 (0, 30)	9 (0, 50)	
Depression (PHQ9)		18			**< 0.001**
Mean (SD)	7.7 (5.9)		5.0 (4.9)	9.2 (5.8)	
Median (IQR)	7.0 (3.0, 11.0)		4.0 (1.0, 8.0)	8.0 (5.0, 13.0)	
PTSD (PCL5)		8			**< 0.001**
Mean (SD)	25 (19)		17 (16)	30 (18)	
Median (IQR)	23 (9, 39)		12 (3, 25)	28 (15, 43)	
Sleep Quality (PSQI)		28			**< 0.001**
Mean (SD)	10.2 (4.8)		7.9 (4.5)	11.4 (4.4)	
Median (IQR)	10.0 (6.0, 14.0)		8.0 (4.0, 11.0)	12.0 (8.0, 15.0)	
Social support (DRRI-2)		2			**< 0.001**
Mean (SD)	39 (8)		40 (8)	38 (8)	
Median (IQR)	40 (34, 45)		42 (36, 47)	39 (33, 44)	
Self-efficacy (GSE)		3			**< 0.001**
Mean (SD)	32.1 (4.8)		33.3 (4.5)	31.4 (4.8)	
Median (IQR)	32.0 (29.0, 36.0)		34.0 (30.0, 37.0)	31.0 (28.0, 35.0)	

^a^Wilcoxon rank sum test; p-value bolded if < 0.05.

^*^Time since last mTBI only applies to participants with positive mTBI histories (N = 1,353).

**Table 6:** Multivariable logistic regression—experience headaches lately yes/no.

	**Multivariable**		
**Characteristic**	**OR** ^a^	**95% CI** ^b^	***p*** **-value** ^c^
Gender			
Male	—	—	
Female	3.57	2.37, 5.48	**< 0.001**
Num of blast TBIs (combat and noncombat)	1.80	1.47, 2.22	**< 0.001**
Num of combat/nonblast TBIs	1.41	1.13, 1.78	**0.003**
Num of noncombat/nonblast TBIs	1.23	1.02, 1.49	**0.035**
Num of months combat deployed	1.23	1.04, 1.45	**0.014**
Controlled blast exposures			
None	—	—	
Minimal (1–9)	1.06	0.76, 1.48	0.7
Light (10–29)	0.98	0.67, 1.44	>0.9
Moderate (30–98)	1.23	0.81, 1.88	0.3
Heavy (99+)	1.10	0.72, 1.66	0.7
OSA high risk (STOP-BANG)	1.13	0.80, 1.62	0.5
Race			
White	—	—	
Black or African American	0.93	0.67, 1.29	0.7
American Indian or Alaska Native	2.35	0.57, 16.1	0.3
Asian	2.63	1.06, 7.22	**0.046**
Other	0.53	0.30, 0.95	**0.030**
Ethnicity			
Not Hispanic or Latino	—	—	
Hispanic or Latino	1.33	0.93, 1.92	0.12
Alcohol Use (AUDIT-C)			
None	—	—	
Moderate	1.27	0.89, 1.81	0.2
Risky	0.87	0.60, 1.26	0.5
HTN			
No	—	—	
Yes	1.20	0.92, 1.57	0.2
Age	0.76	0.62, 0.93	**0.009**
BMI categories			
20-29	—	—	
< 20	1.00	0.31, 3.62	>0.9
>29	1.03	0.79, 1.33	0.8
Depression (PHQ-9 total score)	1.56	1.13, 2.15	**0.007**
PTSD (PCL-5 total score)	1.54	1.07, 2.23	**0.021**
Sleep quality disturbance (PSQI total score)	1.78	1.40, 2.28	**< 0.001**
Social support (DRRI-2 social total)	1.15	0.95, 1.40	0.2
Combat intensity (DRRI-2 combat total)	1.09	0.83, 1.42	0.5
Self-efficacy (GSE total)	1.08	0.87, 1.35	0.5

^a^OR, Odds ratio (expressed as 75th vs. 25th percentile for continuous variables).

^b^CI, Confidence interval.

^c^p-value bolded if < 0.05.

**Table 7:** Multivariable linear regression for HIT-6 total score (multiple *R*^2^ = 0.350).

	**Multivariable**		
**Characteristic**	**Beta** ^a^	**95% CI** ^b^	***p*** **-value** ^c^
Gender			
Male	-	-	
Female	3.4	2.1, 4.8	**< 0.001**
Num of blast TBIs (combat and noncombat)	0.57	0.02, 1.1	**0.043**
Num of combat/nonblast TBIs	0.38	−0.32, 1.1	0.3
Num of noncombat/nonblast TBIs	−0.09	−0.75, 0.58	0.8
Num of months combat deployed	−0.01	−0.59, 0.57	>0.9
Controlled blast exposures			
None	-	-	
Minimal (1–9)	−0.98	−2.3, 0.33	0.14
Light (10–29)	−0.32	−1.8, 1.2	0.7
Moderate (30–98)	−0.71	−2.3, 0.86	0.4
Heavy (99+)	−0.71	−2.2, 0.81	0.4
OSA high risk (STOP-BANG)	0.69	−0.54, 1.9	0.3
Race			
White	-	-	
Black or African American	2.3	1.1, 3.6	**< 0.001**
American Indian or Alaska Native	2.9	−1.5, 7.2	0.2
Asian	−1.0	−4.4, 2.4	0.6
Other	2.4	0.28, 4.5	**0.027**
Ethnicity			
Not Hispanic or Latino	-	-	
Hispanic or Latino	2.0	0.79, 3.3	**0.001**
Alcohol use (AUDIT-C)			
None	-	-	
Moderate	−0.52	−1.8, 0.75	0.4
Risky	−2.3	−3.6, −0.91	**0.001**
HTN			
No	-	-	
Yes	0.53	−0.47, 1.5	0.3
Age	−0.98	−1.8, −0.12	**0.026**
BMI categories			
20–29	-	-	
< 20	0.56	−3.6, 4.8	0.8
>29	−0.33	−1.3, 0.67	0.5
Depression (PHQ-9 total score)	0.67	−0.39, 1.7	0.2
PTSD (PCL-5 total)	4.9	3.6, 6.2	**< 0.001**
Sleep quality disturbance (PSQI total score)	1.4	0.48, 2.3	**0.003**
Social support (DRRI-2 social total)	0.24	−0.47, 0.94	0.5
Combat intensity (DRRI-2 combat total)	0.34	−0.61, 1.3	0.5
Self-efficacy (GSE total score)	−0.70	−1.5, 0.09	0.084

^1^Beta expressed as 75th vs. 25th percentile for continuous variables.

^2^CI, Confidence Interval.

^3^p-value bolded if < 0.05.

In the article, there were errors in the following supplementary tables as published. Supplementary Table 1. *Post-hoc* comparisons HIT-6 Total Sore by Number of mild TBI groups; Supplementary Table 2. Post-hoc comparisons of HIT-6 Impact categories by Number of mild TBI groups; Supplementary Table 3. Prevalence of Headache Lately; Logistic regression sensitivity analysis including only mTBI positive participants (*N* = 1,234); Supplementary Table 4. Headache Impact (HIT6 Total Score); Linear Regression sensitivity analysis including only mTBI positive participants who endorsed HA Lately (*N* = 853).

In the published article, there were errors in the **Abstract**, *Methods* and *Results* sections as published. They should have been written as:

**Methods:** Participants with non-credible symptom reporting were excluded, leaving *N* = 1,674 of whom 81% had positive mTBI histories.

**Results:** In covariate-adjusted analysis, HA prevalence was higher with greater number of blast-related mTBIs (OR 1.81; 95% CI 1.48, 2.23) non-blast mTBIs while deployed (OR 1.42; 95% CI 1.14, 1.79), or non-blast mTBIs when not deployed (OR 1.23; 95% CI 1.02, 1.49).

In the published article, there were errors in the **Methods**, *Participants* section as published. This should have been written as:

For this secondary analysis, all LIMBIC-CENC PLS participants whose enrollment (baseline) assessment data were available at time of dataset extraction were included (*n* = 1,832). …. We also excluded participants with evidence of noncredible symptom reporting based on failing (126) the Mild Brain Injury Atypical Symptom (mBIAS) scale, a validated self-reported measure of symptom reporting credibility in the mTBI population using the developer's recommended cut-point of 8 or higher (Cooper et al., 2011). This left a final analytic sample of 1,674 participants (see Figure 1).

**Figure 1:**
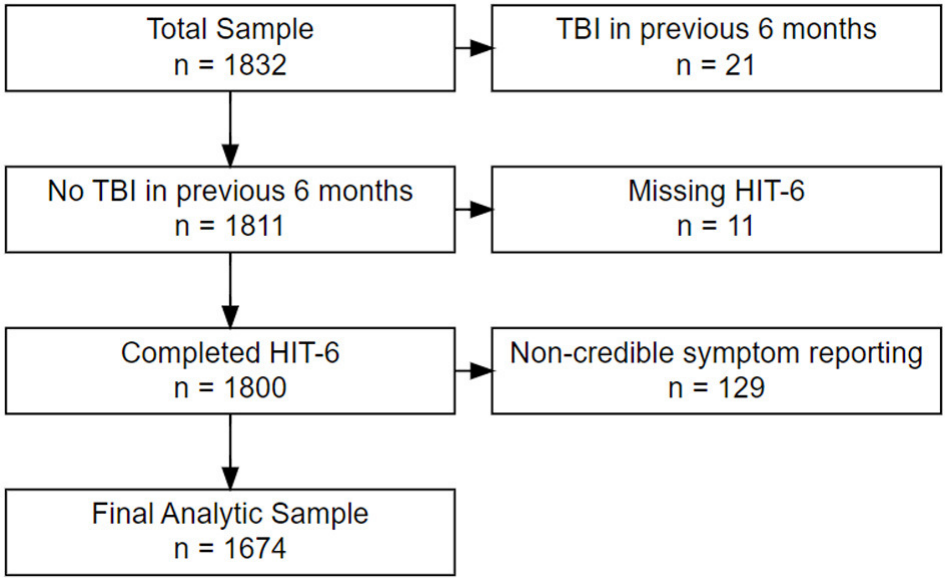
Study sample inclusion flow diagram.

In the published article, there were errors in the **Results** section as published. This should have been written as:

In our final sample of 1,674 combat-exposed current and former SMs, 19% had an entirely negative lifetime mTBI history, 47% had sustained 1-2 mTBIs, and 34% had 3 or more. Rates of positive history across the mTBI mechanism/setting categories were 63% for Combat mTBI(s), 67% for Non-combat mTBI(s), and 37% for Blast-related mTBI(s).

In the published article, there were errors in the **Results**, *HA prevalence and impact across mTBI history groups (0, 1–2, 3*+*)* section as published. This should have been written as:

For example, the rate of severe HA pain sometimes, often or always was 70% for the no TBI group compared to 78% for those with 1-2 or 3+ lifetime mTBIs. (See Table 3 for HIT-6 item #1 *post-hoc* testing; the other post-hoc testing data are available in **Supplementary Tables S1, S2**).

In the published article, there were errors in the **Results**, *Main multivariable regression analyses* section as published. This should have been written as:

For TBI history, the number of lifetime mTBIs of every type was significant, including blast-related (OR = 1.80), Blunt during combat-deployment (OR = 1.41), and Blunt outside of deployment (OR = 1.23). Other significant factors included identifying as female (OR = 3.57), age (0.76), total months combat-deployed (OR = 1.23), and symptoms of depression on PHQ-9 (OR = 1.56), PTSD on PCL-5 (OR = 1.54), and disturbed sleep quality on PSQI (OR = 1.78).

For TBI history, only blast-related mTBIs were significant (Beta 0.57). Blunt-only mTBIs did not reach significance, regardless of contextual type (combat or non-combat). Other factors found significant in the HIT-6 linear regression that were also significant in the HA prevalence logistic regression were female identity (Beta 3.4), younger age (Beta −0.98), PTSD symptoms (Beta 4.9), and reduced sleep quality (Beta 1.4). Demographic characteristics that were significant in the HIT-6 score linear regression model but not the preceding HA prevalence model were Black racial identity (Beta 2.3) and Hispanic/Latino ethnic identity (Beta 2.0) as compared with White/non-Hispanic racial/ethnic identity.

In the published article, there were errors in the Discussion section as published. This should have been written as:

**Discussion**

The overall sample (*n* = 1,674), which included 19% with negative TBI histories, had a HA point prevalence (i.e., HA lately) of 65%.

The covariate-adjusted logistic regression model for HA prevalence (see Table 6) showed higher prevalence with a greater number of any subtype of mTBI (see Table 6), with the nominally highest OR for blast-related mechanism (OR 1.80; 95% CI 1.47, 2.22).

Our large sample, which included 215 females (13%), enabled us to examine their relative risk for HA, a previously understudied research question in the military population due to insufficient numbers of females in most prior HA studies. Our results show that female sex had the nominally highest OR (3.57; 2.37, 5.48) for experiencing HA lately (see Table 6), and had a strong association with higher HA impact (Beta 3.4; 2.1, 4.8; see Table 7).

In the published article, there were errors in the **Discussion**, *Study strengths* section as published. This should have been written as:

Study strengths included our large sample (*n* = 1,674) of individuals with military combat exposure drawn from the LIMBIC-CENC multicenter cohort with rigorously determined lifetime mTBI histories and a large breadth of data available from their comprehensive assessments.

The authors apologize for this error and state that this does not change the scientific conclusions of the article in any way. The original article has been updated.

