# Supplementary material for: Headache among combat-exposed veterans and service members and its relation to mild traumatic brain injury history and other factors: a LIMBIC-CENC study
Source: Front Neurol. 2023 Sep 20;14:1242871. doi: 10.3389/fneur.2023.1242871 (PMC10552781; doi:10.3389/fneur.2023.1242871)
Supplement: Supplementary file 1 [file Table_1.docx]

**Supplementary Tables**

**Supplementary Tables**

Supplementary Table 1.

**Post-hoc comparisons HIT-6 Total Sore by Number of mild TBI groups**

| **Comparison** | **Z** | **P-value** | **Adjusted P-value*** |
| --- | --- | --- | --- |
| 1-2 mTBIs - 3+ mTBIs | 0.5978843 | 0.550 | 1.00 |
| 1-2 mTBIs - No TBI | 3.2022311 | **0.001** | **0.004** |
| 3+ mTBIs - No TBI | 2.7330797 | **0.006** | **0.019** |

* Dunn’s Test

Supplementary Table 2.

**Post-hoc comparisons of HIT-6 Impact categories by Number of mild TBI groups**

| Dimension | Value | Little/None | Some | Substantial | Severe |
| --- | --- | --- | --- | --- | --- |
| No TBI | Residuals | 3.5209458 | 0.2737689 | -0.7434864 | -2.2300233 |
| No TBI | P values | **0.0051600** | 1.00 | 1.00 | 0.3089510 |
| 1-2 mTBIs | Residuals | -0.7957737 | 0.2058492 | -1.2623490 | 1.3118893 |
| 1-2 mTBIs | P values | 1.00 | 1.00 | 1.00 | 1.00 |
| 3+ mTBIs | Residuals | -1.6521587 | -0.4011780 | 1.8059241 | 0.2234695 |
| 3+mTBIs | P values | 1.00 | 1.00 | 0.8511620 | 1.00 |

Supplementary Table 3.

**Prevalence of Headache Lately; Logistic regression sensitivity analysis including only mTBI positive participants (N=1234)**

|  | Univariable | | | Multivariable | | |
| --- | --- | --- | --- | --- | --- | --- |
| **Characteristic** | **OR**^1^ | **95% CI**^1^ | **p-value** | **OR**^1^ | **95% CI**^1^ | **p-value** |
| Time since last TBI (years) | 0.96 | 0.95, 0.98 | <0.001 | 1.00 | 0.98, 1.02 | >0.9 |
| Early HA after TBI | 1.68 | 1.28, 2.21 | <0.001 | 1.26 | 0.91, 1.77 | 0.2 |
| Gender |  |  |  |  |  |  |
| Male | — | — |  | — | — |  |
| Female | 2.52 | 1.62, 4.10 | <0.001 | 3.51 | 2.08, 6.15 | <0.001 |
| Num of blast TBIs (combat and noncombat) | 2.01 | 1.69, 2.41 | <0.001 | 1.67 | 1.35, 2.10 | <0.001 |
| Num of combat/nonblast TBIs | 1.52 | 1.26, 1.86 | <0.001 | 1.33 | 1.04, 1.70 | 0.026 |
| Num of noncombat/nonblast TBIs | 0.99 | 0.84, 1.17 | >0.9 | 1.08 | 0.87, 1.35 | 0.5 |
| Num of months combat deployed | 1.22 | 1.07, 1.41 | 0.005 | 1.11 | 0.93, 1.33 | 0.3 |
| Controlled blast exposures |  |  |  |  |  |  |
| None | — | — |  | — | — |  |
| Minimal (1-9) | 1.04 | 0.76, 1.43 | 0.8 | 1.19 | 0.81, 1.75 | 0.4 |
| Light (10-29) | 1.19 | 0.83, 1.72 | 0.3 | 1.20 | 0.77, 1.88 | 0.4 |
| Moderate (30-98) | 1.44 | 0.98, 2.12 | 0.063 | 1.47 | 0.91, 2.38 | 0.11 |
| Heavy (99+) | 1.74 | 1.21, 2.52 | 0.003 | 1.20 | 0.74, 1.95 | 0.5 |
| OSA high risk | 1.68 | 1.24, 2.31 | 0.001 | 1.29 | 0.87, 1.93 | 0.2 |
| Race |  |  |  |  |  |  |
| White | — | — |  | — | — |  |
| Black or African American | 1.24 | 0.91, 1.72 | 0.2 | 0.98 | 0.67, 1.44 | >0.9 |
| American Indian or Alaska Native | 1.40 | 0.41, 6.33 | 0.6 | 1.33 | 0.31, 9.21 | 0.7 |
| Asian | 3.49 | 0.98, 22.2 | 0.10 | 8.18 | 2.14, 53.9 | 0.007 |
| Other | 0.95 | 0.59, 1.57 | 0.8 | 0.61 | 0.33, 1.16 | 0.12 |
| Ethnicity |  |  |  |  |  |  |
| Not Hispanic or Latino | — | — |  | — | — |  |
| Hispanic or Latino | 1.39 | 1.01, 1.93 | 0.049 | 1.16 | 0.77, 1.76 | 0.5 |
| AUDIT-C |  |  |  |  |  |  |
| 0 | — | — |  | — | — |  |
| 1 | 0.75 | 0.53, 1.06 | 0.10 | 1.11 | 0.73, 1.68 | 0.6 |
| 2 | 0.58 | 0.40, 0.81 | 0.002 | 0.74 | 0.48, 1.13 | 0.2 |
| HTN |  |  |  |  |  |  |
| No | — | — |  | — | — |  |
| Yes | 1.21 | 0.95, 1.55 | 0.13 | 1.10 | 0.81, 1.50 | 0.5 |
| Age | 0.80 | 0.67, 0.95 | 0.012 | 0.84 | 0.64, 1.10 | 0.2 |
| BMI categories |  |  |  |  |  |  |
| 20-29 | — | — |  | — | — |  |
| <20 | 1.27 | 0.36, 5.84 | 0.7 | 0.52 | 0.12, 2.87 | 0.4 |
| >29 | 1.12 | 0.89, 1.42 | 0.3 | 0.90 | 0.67, 1.22 | 0.5 |
| PHQ-9 total | 3.27 | 2.67, 4.04 | <0.001 | 1.45 | 1.01, 2.10 | 0.046 |
| PCL-5 total | 3.97 | 3.15, 5.05 | <0.001 | 1.66 | 1.09, 2.55 | 0.019 |
| PSQI total | 3.32 | 2.72, 4.07 | <0.001 | 1.88 | 1.42, 2.48 | <0.001 |
| DRRI-2 social total | 0.66 | 0.56, 0.78 | <0.001 | 1.07 | 0.86, 1.34 | 0.5 |
| DRRI-2 combat total | 2.06 | 1.69, 2.53 | <0.001 | 1.06 | 0.79, 1.43 | 0.7 |
| GSE total | 0.54 | 0.45, 0.64 | <0.001 | 1.10 | 0.86, 1.42 | 0.5 |
| ^1^OR = Odds Ratio, CI = Confidence Interval | | | | | | |

Supplementary Table 4.

**Headache Impact (HIT6 Total Score); Linear Regression sensitivity analysis including only mTBI positive participants who endorsed HA Lately (N=853)**

|  | Univariable | | | Multivariable | | |
| --- | --- | --- | --- | --- | --- | --- |
| **Characteristic** | **Beta** | **95% CI**^1^ | **p-value** | **Beta** | **95% CI**^1^ | **p-value** |
| Time since last TBI (years) | -0.14 | -0.21, -0.08 | <0.001 | -0.05 | -0.12, 0.02 | 0.2 |
| Early HA after TBI | 0.25 | -0.96, 1.5 | 0.7 | 0.82 | -0.30, 1.9 | 0.2 |
| Gender |  |  |  |  |  |  |
| Male | — | — |  | — | — |  |
| Female | 4.1 | 2.5, 5.8 | <0.001 | 3.4 | 1.8, 5.0 | <0.001 |
| Num of blast TBIs (combat and noncombat) | 0.76 | 0.19, 1.3 | 0.009 | 0.31 | -0.29, 0.90 | 0.3 |
| Num of combat/nonblast TBIs | 0.64 | -0.14, 1.4 | 0.11 | 0.10 | -0.65, 0.85 | 0.8 |
| Num of noncombat/nonblast TBIs | -1.4 | -2.1, -0.63 | <0.001 | -0.42 | -1.1, 0.29 | 0.2 |
| Num of months combat deployed | -0.04 | -0.66, 0.59 | >0.9 | -0.03 | -0.66, 0.60 | >0.9 |
| Controlled blast exposures |  |  |  |  |  |  |
| None | — | — |  | — | — |  |
| Minimal (1-9) | -2.5 | -4.1, -0.91 | 0.002 | -1.4 | -2.8, 0.03 | 0.055 |
| Light (10-29) | -1.4 | -3.2, 0.45 | 0.14 | -0.74 | -2.4, 0.89 | 0.4 |
| Moderate (30-98) | -2.2 | -4.0, -0.34 | 0.020 | -1.1 | -2.8, 0.55 | 0.2 |
| Heavy (99+) | -1.2 | -2.9, 0.45 | 0.2 | -0.70 | -2.3, 0.94 | 0.4 |
| OSA high risk | 0.46 | -0.88, 1.8 | 0.5 | 0.32 | -0.99, 1.6 | 0.6 |
| Race |  |  |  |  |  |  |
| White | — | — |  | — | — |  |
| Black or African American | 3.4 | 1.9, 4.8 | <0.001 | 2.3 | 0.94, 3.7 | <0.001 |
| American Indian or Alaska Native | 2.1 | -3.5, 7.8 | 0.5 | 1.7 | -3.2, 6.5 | 0.5 |
| Asian | -4.6 | -9.0, -0.22 | 0.039 | -0.19 | -4.0, 3.6 | >0.9 |
| Other | 4.4 | 2.0, 6.8 | <0.001 | 2.4 | 0.22, 4.6 | 0.031 |
| Ethnicity |  |  |  |  |  |  |
| Not Hispanic or Latino | — | — |  | — | — |  |
| Hispanic or Latino | 3.4 | 2.0, 4.9 | <0.001 | 1.6 | 0.23, 2.9 | 0.022 |
| AUDIT-C |  |  |  |  |  |  |
| 0 | — | — |  | — | — |  |
| 1 | -3.0 | -4.5, -1.6 | <0.001 | -1.1 | -2.4, 0.26 | 0.11 |
| 2 | -4.7 | -6.2, -3.1 | <0.001 | -2.9 | -4.3, -1.5 | <0.001 |
| HTN |  |  |  |  |  |  |
| No | — | — |  | — | — |  |
| Yes | 0.64 | -0.52, 1.8 | 0.3 | 0.46 | -0.60, 1.5 | 0.4 |
| Age (years) | -1.2 | -2.1, -0.29 | 0.010 | -0.63 | -1.6, 0.40 | 0.2 |
| BMI categories |  |  |  |  |  |  |
| 20-29 | — | — |  | — | — |  |
| <20 | 1.7 | -4.4, 7.8 | 0.6 | 2.5 | -2.9, 8.0 | 0.4 |
| >29 | 0.30 | -0.83, 1.4 | 0.6 | -0.23 | -1.3, 0.85 | 0.7 |
| PHQ-9 total | 4.9 | 4.2, 5.6 | <0.001 | 0.66 | -0.47, 1.8 | 0.3 |
| PCL-5 total | 7.2 | 6.4, 8.0 | <0.001 | 4.9 | 3.5, 6.2 | <0.001 |
| PSQI total | 5.2 | 4.4, 6.1 | <0.001 | 1.2 | 0.23, 2.2 | 0.016 |
| DRRI-2 social total | -2.0 | -2.7, -1.3 | <0.001 | 0.19 | -0.56, 0.93 | 0.6 |
| DRRI-2 combat total | 1.4 | 0.52, 2.3 | 0.002 | 0.13 | -0.88, 1.1 | 0.8 |
| GSE total | -3.7 | -4.5, -2.9 | <0.001 | -0.60 | -1.5, 0.26 | 0.2 |
| ^1^CI = Confidence Interval | | | | | | |
